# Morphological Characteristics and Phylogeny Reveal Six New Species in *Russula* Subgenus *Russula* (*Russulaceae, Russulales*) from Yanshan Mountains, North China

**DOI:** 10.3390/jof8121283

**Published:** 2022-12-07

**Authors:** Hao Zhou, Gui-Qiang Cheng, Qiu-Tong Wang, Mei-Jun Guo, Lan Zhuo, Hui-Fang Yan, Guo-Jie Li, Cheng-Lin Hou

**Affiliations:** 1 College of Life Science, Capital Normal University, Xisanhuan Beilu 105, Beijing 100048, China; 2 College of Horticulture, Hebei Agricultural University, Baoding 071001, China; 3 Key Laboratory of Vegetable Germplasm Innovation and Utilization of Hebei, Collaborative Innovation Center of Vegetable Industry in Hebei, Baoding 071001, China

**Keywords:** *Russulaceae*, new taxa, edible fungi, taxonomy

## Abstract

Species of the genus *Russula* are key components of ectomycorrhizal ecosystems worldwide, some of which are famous edible fungi. Although many new species have been described in China, their diversity in North China is still poorly known. Based on the morphology observation of specimens and molecular phylogenetic analyses, combined with the current classification frame of *Russula*, six new species of *Russula* subgenus *Russula* are proposed from the Yanshan Mountains in northern Beijing and northern Hebei Province of China in this study: viz. *Russula miyunensis* (subsection *Chamaeleontinae*), *R*. *plana* (subsection *Chamaeleontinae*), *R. sinoparva* (subsection *Puellarinae*), *R. sinorobusta* (subsection *Puellarinae*), *R. subversatilis* (subsection *Roseinae*), and *R. yanshanensis* (subsection *Puellarinae*). This is the first report of the species of *Russula* subgenus *Russula* from the Yanshan Mountains. This study enriches the species diversity of *Russula* in North China and provides new data support for the systematic study of *Russula* in subsequent research, including research and development on edibility.

## 1. Introduction

*Russula* Pers. (*Russulaceae*, *Russulales*, *Agaricomycetes*, and *Basidiomycota*) was established in 1796, which is one of the most abundant genera, including at least 2000 species [1,2]. This genus is mainly characterized by colorless to multi-colored pileus, amyloid warty basidiospores, abundant spherocytes in a heteromerous trama, an absence of latex, and hyphae without clamp connections [3,4,5]. *Russula* is a large genus of ectomycorrhizal (ECM) fungi that are found in all common ecosystems, such as broad-leaved forest, coniferous forest, mixed coniferous, broad-leaved forest, or scrubland [6,7,8]. Furthermore, some members of *Russula* not only play an important role in ecology by symbiotic with a variety of plants but also serve as a food source for many animals, including humans. Some species of *Russula*, e.g., *Russula delica* Fr., *Russula griseocarnosa* X.H. Wang, Zhu L. Yang, & Knudsen, *Russula nigricans* Fr. et al., are famous edible fungi and important commercial trade goods in the world [7,9,10,11,12]. According to recent statistics on the diversity of Chinese edible macrofungi resources, there are about 70 edible species in China [12].

The previous classification system for *Russula* was based on morphology, e.g., pileus color, spore print, and spore. Miller and Buyck et al. first used phylogenetic analysis of nrITS loci to compare with the previous classification system of *Russula* in Europe, resulting in 78 *Russula* species forming six clades with higher supported values on the phylogenetic tree [13]. Buyck et al. [1,2] demonstrated that *Russula* was one of four monophyletic groups in non-corticoid *Russulaceae* and was divided into eight subgenera by multi-locus phylogenetic studies [1,14].

The subgenus *Russula* Pers. is a species-rich subgenus of *Russula*, which is morphologically characterized mainly by a great variation of basidiocarp size, pileus thick to extremely thin fleshed; stipe abnormally annulate gills unusually equal or lamellulae; spore print white to yellow; spores with amyloid suprahilar spot. Phylogenetically, this subgenus is divided into two parts: a core and a crown clade [1,2].

The first record of *Russula* in China is *Russula alutacea* (Fr.) Fr. from Tibet and Sichuan Province [15]. So far, about 190 species have been recorded in China [6,8,16,17,18,19,20,21,22,23,24,25,26,27,28,29,30,31], and nearly 20 species belong to the subgenus *Russula* [18,23,26,32,33,34,35,36,37]. As a subgenus with the most species in the genus *Russula*, its members are also widely distributed all over the world.

The Yanshan Mountains (115°–119°47′ E, 39°40′–41°20′ N) are located in North China and have a warm temperate continental monsoon climate. This region is known for its high plant diversity. The main forest types of this region are deciduous broad-leaved forest and mixed coniferous and broad-leaved forest. Dominant ectomycorrhizal trees in this region include *Pinus tabuliformis* Carr, *Betula* spp., *Quercus* spp. and *Abies* (Mill.) spp. The Yanshan Mountains have an annual precipitation of approximately 350–700 mm, and their altitude ranges from 200 to 2200 m [37,38]. Until the present study, records about the *Russula* species in this area were very few [39].

In this study, six new species of the subgenus *Russula* crown clade from the Yanshan Mountains were described based on multi-locus phylogenetic analyses and detailed macro- and micromorphological data. The aims of this study are to identify the taxonomic status and phylogenetic position of new species, to establish a comprehensive database on the diversity of macrofungal in North China, especially the status of *Russula* in the Yanshan Mountains, and to use this as a basis for promoting research on macrofungal diversity and edible *Russula* species in this region.

## 2. Materials and Methods

### 2.1. Sampling and Morphological Observations

Specimens were collected from 2017 to 2021. Fresh specimens were photographed in the field, and characteristics such as color, odor, and viscosity were noted. Specimens were dried with a Dorrex dryer at 45 °C and deposited in the Herbarium of the College of Life Science, Capital Normal University, Beijing, China (BJTC). Macroscopic characteristics were recorded from fresh specimens. Microscopic characteristics were observed from thin sections of dried material mounted in 3% KOH or sterilized water. Congo Red (1%) was used to make the structures more visible. Melzer’s reagent was used to test the amyloid reaction of the spores [40]. All tissues were also examined in cresyl blue to verify the presence of ortho- or metachromatic reactions, as explained in Buyck [1]. Cystidia contents were examined in sulfovanillin (SV) solution [40]. Microscopic structures (e.g., basidiospores, basidia, cystidia) were observed and measured using a light microscope (Olympus DP71, Tokyo, Japan) and Image Pro Plus 6.0. The basidiospore structures were further observed under a field emission scanning electron microscope (SEM, Hitachi S-4800, Tokyo, Japan), digital cameras (Olympus U-TV0.5XC-3, Tokyo, Japan), and measuring software (Image Pro Plus 6.0). Basidiospore measurements were presented as (Min–) AV-SD–AV–AV + SD (–Max), where Min is the minimum value, Max is the maximum value, AV is the average value, SD is the standard deviation, and Q represents the length/width ratio of the basidiospores [8]. Statistics for the microscopic characteristics (e.g., basidiospores and basidia) were based on 30 measurements per specimen. The descriptive terms follow Adamčík et al. [2]. In this study, color codes were used from the reference website colorhexa (https://www.colorhexa.com (accessed on 8 September 2022)).

### 2.2. DNA Extraction and Sequencing

**Figure 1:**
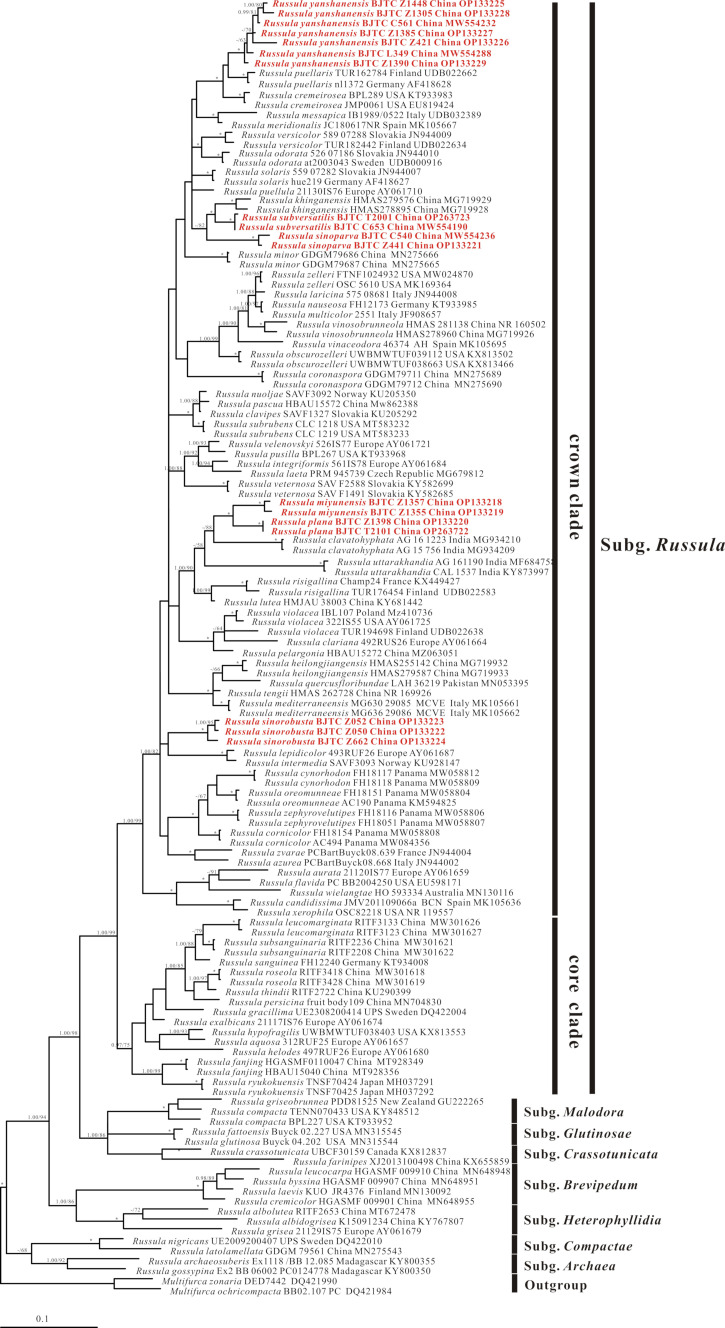
The nrITS phylogenetic tree obtained from the Bayesian analysis. Numbers above branches represent strongly and moderately support (pp ≥ 0.95 and/or MLB ≥ 50%). Numbers above branches are Bayesian posterior probability (pp) values and maximum likelihood bootstrap (MLB). The red font indicates the position of newly obtained sequences. Accession numbers of sequences information used are indicated on the figure. Asterisks (*) denote branches with pp = 1.00, MLb = 100%.

DNA extraction was achieved via the M5 Plant Genomic DNA Kit (Mei5 Biotechnology, Co., Ltd., Beijing, China). The DNA obtained was dissolved in 1 × TE buffer/sterile water and stored at −20 °C for later use. The PCR amplifications were performed in a Bio-Rad S1000^TM^ Thermal Cycler (Bio-Rad Laboratories, Inc, Hercules, CA, USA). The primer set nrITS 1f/nrITS 4 was used to amplify for rDNA ITS region [41], T LR0R/LR5 for the large subunit nuclear ribosomal DNA (nuLSU rDNA) region [42], MS1/MS2 for the ribosomal mitochondrial small subunit (mtSSU) region [41], RBP2-6f/RBP2-7r for the second largest subunit of RNA polymerase II (*rpb2*) region [43] and tef1F/tef1R for the second largest subunit of transcription elongation factor 1-alpha (*tef-1α*) region [44], respectively. The PCR volume was 25 μL, and the detailed composition was described by Zhou et al. [39]. PCR amplification conditions for nrITS and nrLSU refer to Li et al. [25]. PCR amplification conditions for mtSSU and *rpb2* refer to Song et al. [8]. PCR amplification conditions for *tef-1α* refer to Morehouse et al. [44]. DNA sequences were sequenced by Zhongkexilin Biotechnology, Co., Ltd., Beijing, China. Newly obtained sequences in this study were submitted to the NCBI GenBank database (https://submit.ncbi.nlm.nih.gov/ (accessed on 20 July 2022)). Accession numbers of sequences used for phylogenetic analyses are provided in Figure 1 and Table 1. 

### 2.3. Molecular Phylogenetic Analyses

Raw reads of the generated DNA sequences were used to obtain consensus sequences using SeqMan v.7.1.0 (DNASTAR Inc., Madison, WI, USA). All sequences (nrITS, nrLSU, *rpb2*, *tef-1α* and mtSSU) were analyzed using MAFFT v.6 and manually trimmed using MEGA 6 [45]. All reference sequences of subgenus *Russula* of dataset were chosen for phylogenetic analyses based on previous studies and GenBank database in NCBI.

**Figure 2:**
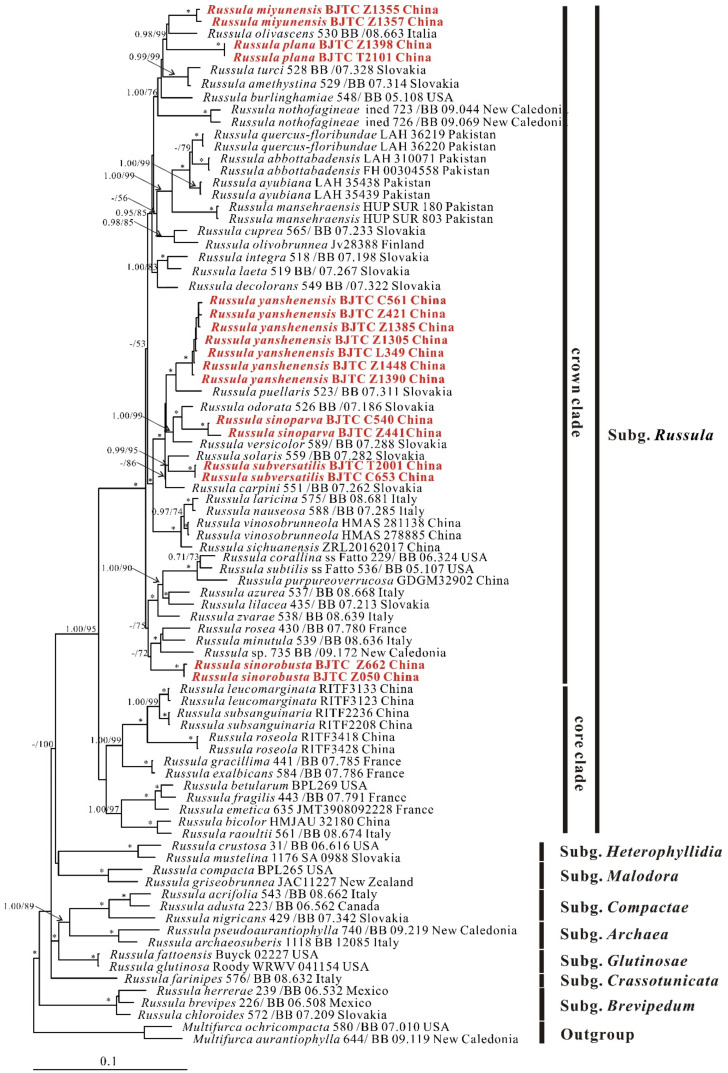
The nrLSU-*rpb2*-*tef-1α*-mtSSU multi-locus phylogenetic tree obtained from the Bayesian analysis. Numbers above branches represent strongly and moderately support (pp ≥ 0.95 and/or MLB ≥ 50%). Numbers above branches are Bayesian posterior probability (pp) values and maximum likelihood bootstrap (MLB). The red font indicates the position of newly obtained sequences. Accession numbers of sequences information used are indicated in Table 1. Asterisks (*) denote branches with pp = 1.00, MLb = 100%.

The nrLSU-*rpb2*-*tef-1α*-mtSSU multi-locus phylogenetic analysis included 83 ingroup samples, which were used to analyze the phylogenetic position of our specimens in the genus *Russula*. Moreover, the nrITS phylogenetic analysis included 129 ingroup samples, which were used to analyze the relationships among our collections and other species in the subgenus *Russula*. All reference sequences of subgenus *Russula* of the dataset were chosen for phylogenetic analyses based on previous studies and the GenBank database in NCBI and UNITE (accession number in Table 1 and Figure 2). *Multifurca aurantiophylla* (Bills & O.K. Mill.) Buyck and V. Hofst. (644/BB 09.119), *Multifurca ochricompacta* (Bills & O.K. Mill.) Buyck & V. Hofst. (BB02.107), *M. ochricompacta* (580/BB 07.010), *Multifurca zonaria* (Buyck & Desjardin) Buyck & V. Hofst. (DED7442), and *Multifurca* were outgroup taxa referring to Buyck et al. [1].

The maximum likelihood (ML) gene trees were estimated using the RAxML 7.4.2 Black Box software [38,46,47,48,49]. ML analysis used a GTR locus substitution model by running 1000 bootstrap replicates with all default settings parameters [50]. Bayesian inference (BI) phylogenetic analysis was performed using MrBayes v.3.1.2 [51]. Branch supports were calculated using a bootstrapping (BS) method with 1000 replicates [52]. Bayesian inference (BI) analysis was performed by the Markov chain Monte Carlo (MCMC) algorithm [53]. MrModeltest v. 2.3 was used to estimate the best model for Bayesian inference (GTR + I + G for nrITS, nrLSU, *rpb2*, and mtSSU; SYM + I + G for *tef-1α*) [51]. Two MCMC chains were run from the random trees for 10,000,000 generations and stopped when the mean standard deviation of split frequencies fell below 0.01. Trees were saved once every 1000 generations by the default settings. The first 25% of trees were discarded as the burn-in phase of each analysis. In the remaining trees, branches with significant Bayesian posterior probabilities were estimated, it has relatively stable topologies, and clades with high Bayesian posterior probability (pp) values can also illustrate relative relationships between species [54]. ML bootstrap support (BS) ≥ 50% and Bayesian posterior probability (PP) ≥ 0.95 were shown on the nodes in Figure 1 and Figure 2.

## 3. Results

### 3.1. Phylogenetic Analyses

The nrITS phylogenetic analysis included 129 ingroup samples, *M. zonaria* (DED7442) and *M. ochricompacta* (BB02.107) were used as the outgroups. The dataset of nrITS loci comprised 611 characters including alignment gaps. The best Bayesian tree is shown in Figure 1. The nrLSU-*rpb2*-*tef-1α*-mtSSU multi-locus phylogenetic analysis included 83 ingroup samples, *M. ochricompacta* (580/BB 07.010) and *M. aurantiophylla* (644/BB 09.119) were used as the outgroups. The dataset of multi-locus comprised 2511 characters including alignment gaps. The nrLSU-*rpb2*-*tef-1α*-mtSSU dataset was analyzed by ML analysis and BI analysis. Phylogenetic analysis generated topologies from ML analysis and BI analysis were almost identical, and the Bayesian tree are shown in Figure 2. 

The nrLSU-*rpb2*-*tef-1α*-mtSSU and nrITS phylogenetic analyses revealed that the subgenera proposed by Buyck et al. [1] were well-supported with significant Bayesian posterior probability (PP) values and maximum likelihood bootstrap (MLB). Sequences of our collections all fell into the *Russula* subgenus *Russula* crown clade and formed six new lineages (marked in red and bolded in Figure 1 and Figure 2) with significant support. Thus, they were considered as six distinct clades and described as new species in this paper, i.e., *Russula miyunensis*, *R. plana*, *R. sinoparva*, *R. sinorobusta*, *R. subversatilis*, and *R. yanshanensis*.

The nrLSU-*rpb2*-*tef-1α-*mtSSU multi-locus phylogenetic analysis showed that two sequences of the new species *Russula sinoparva* (BJTC C540, BJTC Z441) were supported as one clade (pp = 1.00, MLB = 100%) and clustered with *Russula odorata* Romagn. *Russula subversatilis* (BJTC C653, BJTC T2001) formed one clade (pp = 0.99, MLB = 99%) together with *Russula solaris* Ferd. & Winge. Sequences of seven specimens of *Russula yanshanensis* (BJTC C561, BJTC Z421, BJTC Z1385, BJTC Z1305, BJTC L349, BJTC Z1448, and BJTC Z1390) were clustered together, forming a completely supported clade (pp = 1.00, MLB = 100%), *R. yanshanensis* clustered with *Russula puellaris* Fr. and formed a sister clade in the phylogenetic tree. Two new clades of *Russula miyunensis* (BJTC Z1355, BJTC Z1357) and *Russula plana* (BJTC Z1398, BJTC T2101) clustered with *Russula olivascens* Fr. formed a group (pp = 0.98, MLB = 99%). Sequences of three specimens of *Russula sinorobusta* (BJTC Z052, BJTC Z050, and BJTC Z662) clustered into a branch, with high support values (pp = 1.00, MLbs = 100%), and the branch further clustered a clade with *Russula minutula* Velen., *Russula rosea* Pers. and *Russula* sp. (735/BB 09.172) with moderate support. 

The nrITS phylogenetic analysis showed similar topologies to that of multi-locus phylogenetic tree, and sequences of our collections also formed six strong support end branches. Notebly, *R. subversatilis* and *R. sinoparva* were clustered together with *R. khinganensis* G.J. Li & R.L. Zhao. *Russula miyunensis* and *R. plana* formed a well-supported clade. *Russula sinorobusta* formed a clade clustered with *Russula lepidicolor* Romagn. and *Russula intermedia* P. Karst. but without support values. 

### 3.2. Taxonomy

Based on phylogenetic analyses and morphology, six new species of *Russula* subgenus *Russula* from the Yanshan Mountains were recognized and described in this study.

***Russula miyunensis* C. L. Hou, H. Zhou,** & **G. Q. Cheng, sp. nov.**

**Figure 3:**
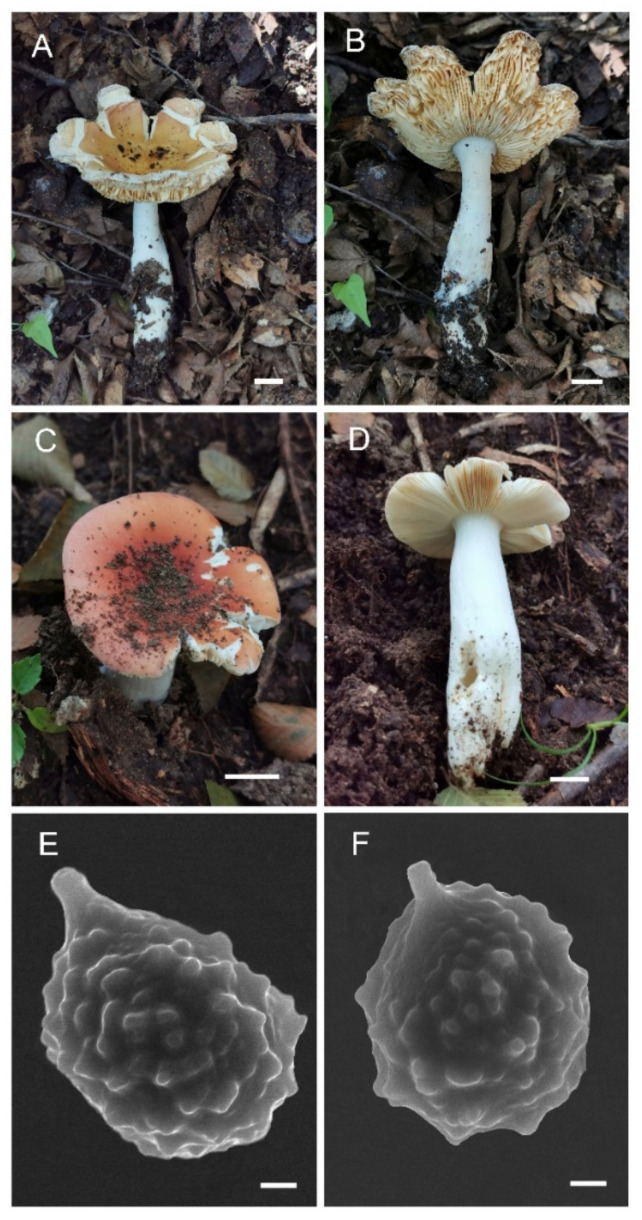
*Russula miyunensis* (BJTC Z1355). (**A**–**D**) Basidiomata. (**E**,**F**) Basidiospores. Scale bar: (**A**–**D**) = 10 mm, (**E**,**F**) = 1 μm.

**Figure 4:**
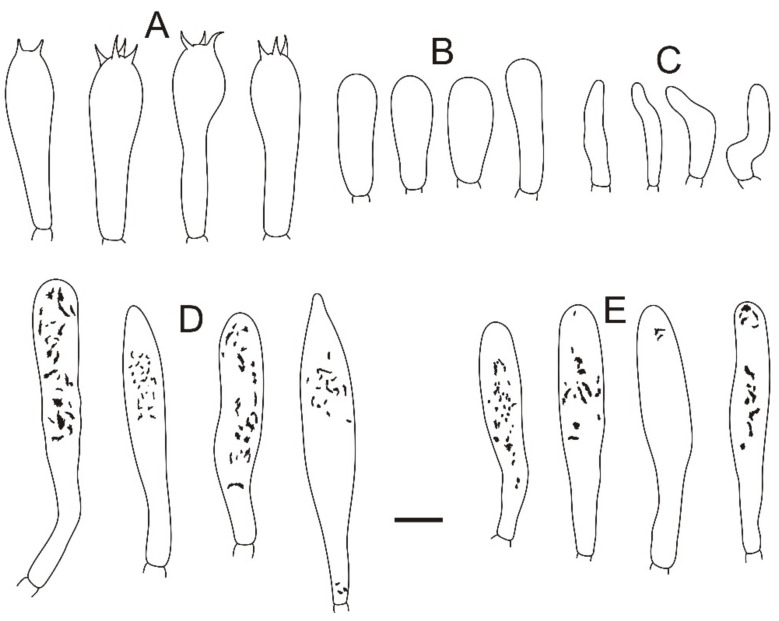
Microscopic features of *Russula miyunensis* (BJTC Z1355). (**A**) Basidia. (**B**) Basidiola. (**C**) Marginal cells. (**D**) Hymenial cystidia on lamellae sides. (**E**) Hymenial cystidia on lamellae edges. Scale bar: 10 μm.

**Figure 5:**
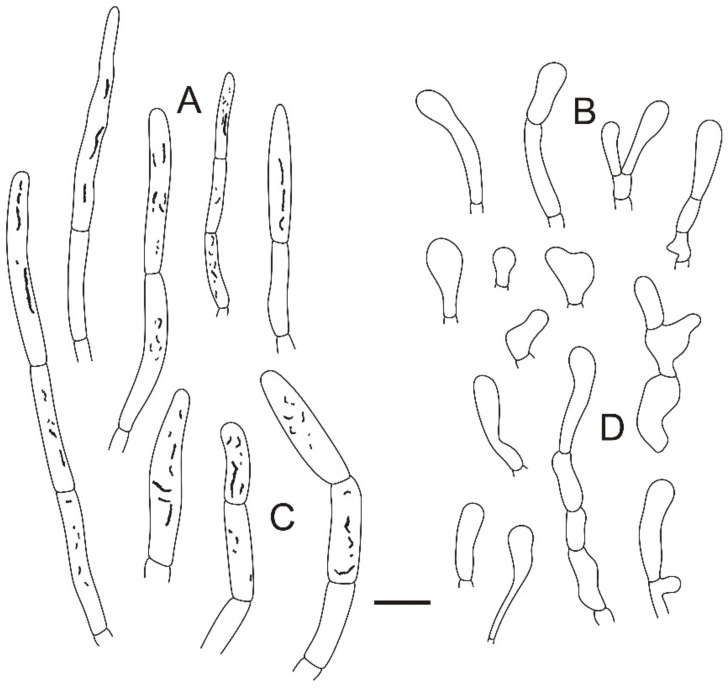
Microscopic features of *Russula miyunensis* (BJTC Z1355). (**A**) Primordial hyphae near the pileus margin. (**B**) Hyphal terminations near the pileus margin. (**C**) Primordial hyphae near the pileus center. (**D**) Hyphal terminations near the pileus center. Scale bar: 10 μm.

**Figure 6:**
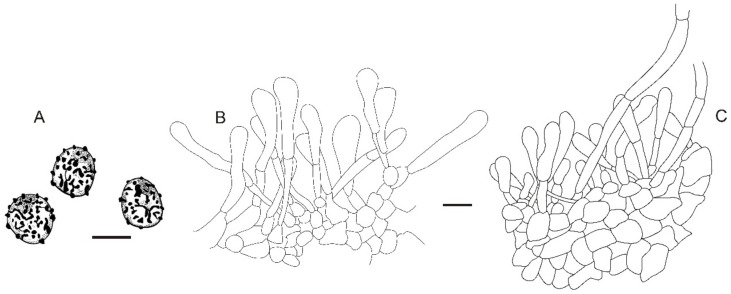
Microscopic features of *Russula miyunensis* (BJTC Z1355). (**A**) Basidiospores. (**B**) Hyphal terminations near the pileus margin. (**C**) Hyphal terminations near the pileus center. Scale bar: (**A**) = 5 μm; (**B**,**C**) = 10 μm.

Figure 3, Figure 4, Figure 5 and Figure 6.

**MycoBank: MB 845047**

**Diagnosis:***Russula miyunensis* is diagnosed by small to big-sized basidiomata, light pink or grayish-yellow, central deep red to deep brown pileus, basidiospores ornamented with amyloid warts, and more or less chain-like, suprahilar spot obvious, longer basidia, shorter terminal cells near the pileus margin, pileocystidia without color change in sulfovanillin. Morphologically, *R. miyunensis* is similar to *Russula olivascens* Fr. and *Russula clavatohyphata* R.P. Bhatt, A. Ghosh, Buyck, & K. Das, but pileus of *R. miyunensis* has severely cracked when mature.

**Holotype:** CHINA, Beijing, Miyun District, Heilongtan, 40°33′42″ N, 116°46′20″ E, alt. 381 m, 29 August 2021, coll. C.-L.H., H.Z. and G.-Q.C. (BJTC Z1355).

**Etymology:** The epithet “*miyunensis”* referred to the locality “Miyun District” where the type specimen was collected.

**Basidiomata:** small to big size, pileus 32–135 mm in diameter, initially hemispherical when young, applanate with depressed in the center when mature, large pieces crack near the margin in age, smooth, peeling to 1/3 of the radius, margin light pink (#d69188) or grayish-yellow (#cec7a7), sometimes light yellow (#d59a6f), central deep red (#af4d43) to deep brown (#623f2d). **Lamellae:** white (#ffffff) to light yellow-brown (#ffffed), adnate, lamellulae absent, hardly forked. **Stipe:** 52–89 × 25–34 mm, white (#ffffff), sometimes with brownish (#a52a2a) on the base, cylindrical, inflated toward base, hollow, smooth. **Context:** 8–34 mm thick in half of the pileus radius, white (#ffffff), without color change when bruised. **Spore print:** not observed.

**Basidiospores:** (6.7–)6.9–7.4–7.9(–8.5) × (5.7–)6.1–6.4–6.7(–7) μm, [Q = (1.03–)1.09–1.17–1.23(–1.31)], globose to broadly ellipsoid, ornamentation of relatively small, dense [(5–)6–9(–10)] in a 3 μm diameter circle] amyloid warts, 0.3–0.7 μm high, occasionally with isolated warts, occasionally fused in pairs or triplets chains [0–2 in the circle], occasionally to frequently connected by short, fine line connections [1–3(–4) in the circle], suprahilar spot obvious, amyloid. **Basidia:** (35–)36.2–38.5–40.8(–43) × (10–)10.4–11.9–13.4(–15) μm, 2–4-spored, clavate, with particles and oil droplets, basidiola clavate or subcylindrical, ca. 7–12 μm wide. **Hymenial cystidia:** dispersed, ca. 400–600/mm^2^. **Hymenial cystidia on lamellae sides:** (45–)50.3–59.7–69.1(73–) × (10–)9.7–11–12.3(–13) μm, thin-walled, clavate, subfusiform or lanceolate, apically mainly obtuse, sometimes mucronate, often with 2–5 μm long appendage; contents with heteromorphous and granulose, turning light brown (#a52a2a) in SV. **Hymenial cystidia on lamellae edges:** smaller to hymenial cystidia on lamellae sides, (42–)44.4–50.2–56(–60) × (6.3–)7.4–8.5–9.6(–10) μm, clavate or subfusiform, apically often obtuse, sometimes with 2–3 μm long appendage, refracted inclusions, contents with heteromorphous-granulose, distribute mainly in the middle, relatively less, turning light brown (#a52a2a) in SV. **Marginal cells:** (15–)17.5–20.9–24.3(–27) × (3.0–)3.2–4.3–5.3(–6.4) μm, subcylindrical, occasionally flexible.

**Pileipellis:** orthochromatic in cresyl blue, sharply delimited from the underlying context, 35–60 μm deep, single-layered, weakly gelatinized, composed of relatively dense, intricate, horizontally oriented near the context, 2–7-μm-wide hyphae. Acid-resistant incrustations are present but mainly distinct only on subterminal cells of primordial hyphae, occasionally on terminal cells. Hyphal terminations near the pileus margin occasionally branched, sometimes flexuous, thin-walled; terminal cells (10.4–)12.9–18.7–24.5(–32.2) × (4.0–)4.8–5.7–6.6(–7.1) μm, mainly cylindrical, clavate, subfusiform or irregular shape, apically obtuse, occasionally attenuated, constricted or inflated, a few forked, subterminal cells often wider, ca. 2–8 μm wide, sometimes branched; hyphal terminations near the pileus center similar to those near the pileus margin; terminal cells (13–)15.8–19.7–23.6(–25) × (3.2–)3.7–4.8–5.9(–7.1) μm, cylindrical, clavate, subfusiform or irregular shape, apically obtuse, occasionally constricted or inflated, a few forked; subterminal cells often wider, sometimes branched, ca. 2–6 μm wide. Primordial hyphae near the pileus margin are always 2–3-celled, sometimes one-celled, thin-walled, terminal cells (20–)23.8–37.7–51.5(–71) × (4.0–)4.7–5.5–6.3(–7.2) μm, cylindrical or fusiform, apically usually obtuse, sometimes constricted; contents with heteromorphous-crystalline or banded, no color change in SV. Primordial hyphae near the pileus center are often smaller, always 2–3-celled, thin-walled, terminal cells (14–)23.2–33.1–43 (–48) × (3.4–)4.2–5.1–6(–8.1) μm, cylindrical or subfusiform, apically typically obtuse or occasionally attenuated, contents with heteromorphous-crystalline or banded. **Cystidioid hyphae or oleiferous hyphae:** not observed.

**Habit and habitat:** Scattered in broad-leaved forests of *Carpinus turczaninowii* Hance.

**Additional specimens examined:** CHINA, Beijing, Miyun District, Heilongtan, 40°33′42″ N, 116°46′20″ E, alt. 384 m, 29 August 2021, coll. C.-L.H., H.Z. and G.-Q.C. (BJTC Z1357).

**Notes:***Russula miyunensis* belongs to subsection *Chamaeleontinae* Singer. On the phylogenetic tree, *R. miyunensis* is closely related to *R. olivascens* and *R. plana* (Figure 1). Morphologically, *R. olivascens* has light green-yellow pileus and bigger basidia (39–52 × 12–15 μm) than *R. miyunensis; Russula plana* has a small size, brick-red to deep red pileus, stipe with a pink tone, shorter basidia (23.4–33.2 × 12.1–15.9 μm), hymenial cystidia on lamellae sides turning light brown in SV. Moreover, the similarity of nrITS sequences with *R. plana* is 91.19% (coverage 99%) [55]. *Russula miyunensis* is similar to *R. uttarakhandia* A. Ghosh & K. Das, without molecular data, but *R. uttarakhandia* has a yellow to grayish-yellow color in the pileus middle, smaller basidia (28.5–37 × 12–15.6 μm), hymenial cystidia on lamellae edges without color change in SV, two-layered pileipellis, the absence of hymenial cystidia on lamellae edges, longer terminal cells of near the pileus margin (25–37 × 4–6 μm) [56].

***Russula plana* C. L. Hou, H. Zhou,** & **G. Q. Cheng, sp. nov.**

**Figure 7:**
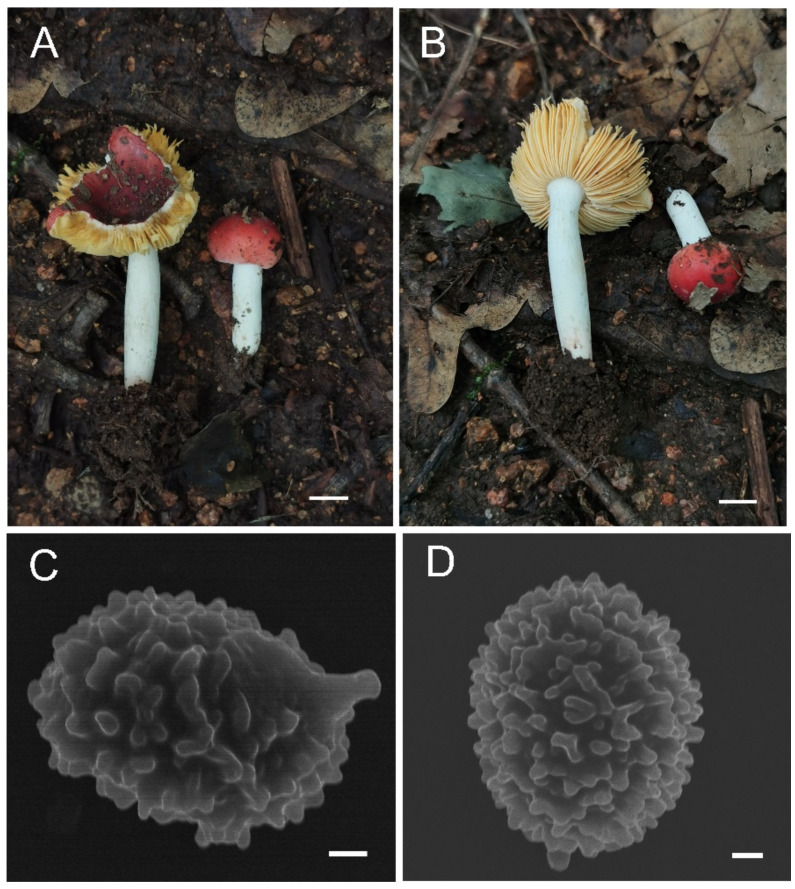
*Russula plana* (BJTC Z1398). (**A**,**B**) Basidiomata. (**C**,**D**) Basidiospores. Scale bar: (**A**,**B**) = 10 mm, (**C**,**D**) = 1 μm.

**Figure 8:**
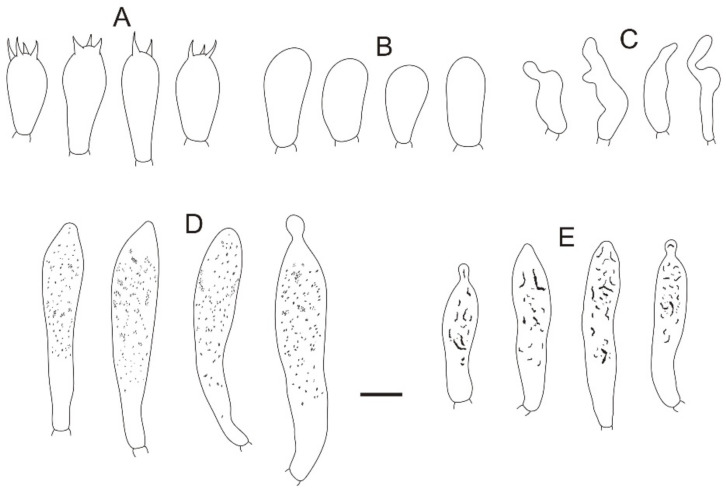
Microscopic features of *Russula plana* (BJTC Z1398). (**A**) Basidia. (**B**) Basidiola. (**C**) Marginal cells. (**D**) Hymenial cystidia on lamellae sides. (**E**) Hymenial cystidia on lamellae edges. Scale bar: 10 μm.

**Figure 9:**
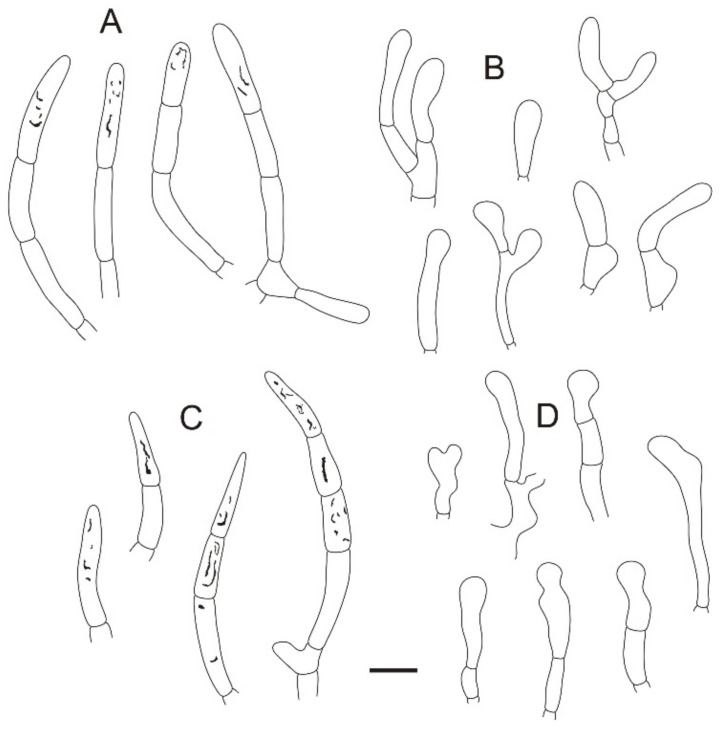
Microscopic features of *Russula plana* (BJTC Z1398). (**A**) Primordial hyphae near the pileus margin. (**B**) Hyphal terminations near the pileus margin. (**C**) Primordial hyphae near the pileus center. (**D**) Hyphal terminations near the pileus center. Scale bar: 10 μm.

**Figure 10:**
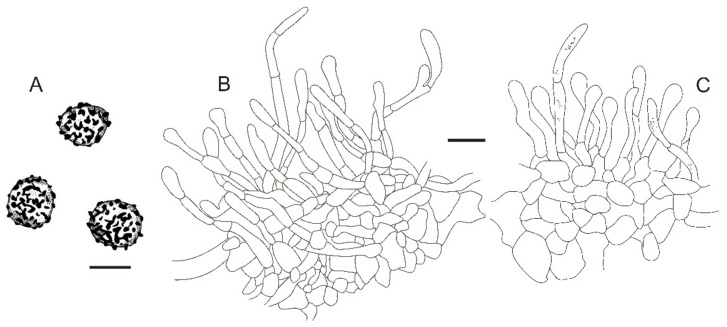
Microscopic features of *Russula plana* (BJTC Z1398). (**A**) Basidiospores. (**B**) Hyphal terminations near the pileus margin. (**C**) Hyphal terminations near the pileus center. Scale bar: (**A**) = 5 μm; (**B**,**C**) = 10 μm.

Figure 7, Figure 8, Figure 9 and Figure 10.

**MycoBank: MB 845049**

**Diagnosis:***Russula plana* is diagnosed by small-sized basidiomata, brick-red to deep red pileus, large basidiospores ornamented with amyloid warts or spines, suprahilar spot large, smaller basidia, longer terminal cells near the pileus margin, pileocystidia without color change in sulfovanillin. *Russula plana* and *Russula clavatohyphata* P. Bhatt, A. Ghosh, Buyck, and K. Das have similar morphological characteristics, but *R. plana* has brick-red to deep red pileus and small basidia and bigger basidiospores. 

**Holotype:** CHINA, Beijing, Miyun District, Sileng Mountain, 40°28′22″ N, 117°6′17″ E, alt. 722 m, 30 August 2021, coll. C.-L.H., H.Z. and G.-Q.C. (BJTC Z1398).

**Etymology:** The epithet “*plana*” refers to the flat pileus of the basidiomata after maturity.

**Basidiomata:** small size, pileus 19–43 mm in diameter, initially hemispherical when young, applanate with depressed in the center when mature, recurved in age, smooth, sticky when wet, peeling to 1/5 of the radius. brick-red (#c62d42) to deep red (#622f30), sometimes red (#ff1a1a), margin light pink (#d69188). **Lamellae:** cream (#ffffff), yellow (#ffffed) in age, adnate to adnexed, lamellulae absent, hardly forked. **Stipe:** 23–41 × 6–13 mm, white (#ffffff), cylindrical, smooth, firm. **Context:** 5–10 mm thick in half of the pileus radius, white (#ffffff), without color change when bruised. **Spore print:** not observed.

**Basidiospores:** (6.8–)7.2–7.9–8.6(–9.9) × (5.8–)6.1–6.7–7.3(–8.3) μm, [Q = (1.02–)1.09–1.18–1.27(–1.44)], subglobose to broadly ellipsoid, ornamentation of relatively of small, dense [(5–)7–9(–10)] in a 3 μm diameter circle] amyloid warts or spines, 0.3–0.8 μm high, occasionally with isolated verrucous, occasionally to frequently fused in pairs or short chains [(0–)1–3(–4) in the circle], occasionally to frequently connected by short, fine line connections [(0–)1–3(–4) in the circle], suprahilar spot large, amyloid. **Basidia:** (22–)23.4–28.3–33.2(–38) × (10–)12.1–14–15.9(–18) μm, 2–4-spored, mainly clavate, sometimes broadly ellipsoid, with particles and oil droplets, basidiola broadly ellipsoid, clavate or subcylindrical, ca. 8–15 μm wide. **Hymenial cystidia:** dispersed, ca. 500–650/mm^2^. **Hymenial cystidia on lamellae sides:** (42–)48.2–57.1–66(–70) × (8.1–)9–10.4–11.8(–13) μm, clavate or subfusiform, apically mainly obtuse, often with 3–5 μm long appendage, thin-walled; contents with heteromorphous or granulose, turning grayish-red (#8b0000) in SV. **Hymenial cystidia on lamellae edges:** smaller to hymenial cystidia on lamellae sides, (36–)39.2–43.3–47.4(–50) × (6.5–)7.3–8.4–9.5(–10) μm, clavate or subfusiform, sometimes subulate, apically often obtuse or constricted, sometimes with 2–5 μm long appendage, refracted inclusions, contents with heteromorphous or granulose, turning ash black (#080808) in SV.

**Marginal cells:** (15–)20.2–25.5–30.8(–33) × (3.8–)3.1–4.8–5.7(–6.4) μm, subfusiform or irregular shape, sometimes flexible. **Pileipellis:** orthochromatic in cresyl blue, sharply delimited from the underlying context, 80–120 μm deep, single-layered, weakly gelatinized, relatively dense, intricate, horizontally oriented near the context, 4–9 μm wide hyphae. Acid-resistant incrustations present, distinct on the terminal or subterminal cells of primordial hyphae, occasionally on terminal cells. Hyphal terminations near the pileus margin occasionally branched, sometimes flexuous, thin-walled; terminal cells (13–)13.4–17.5–21.6(–26) × (3.8–)4.2–5.2–6.2(–7.8) μm, cylindrical, clavate, subfusiform or irregular shape, apically mainly obtuse, sometimes constricted or inflated, less forked, subterminal cells ca. 3–5 μm wide, occasionally branched; hyphal terminations near the pileus center similar to those near the pileus margin; terminal cells (8.4–)12–18–24(–35) × (3.0–)3.7–4.3–4.9 μm, cylindrical, clavate, subfusiform, subulate or irregular shape, apically obtuse, sometimes constricted or inflated; subterminal cells often wider, occasionally branched, ca. 3–6 μm wide. Primordial hyphae near the pileus margin are always single-celled, sometimes 2–3 celled, terminal cells (11–)14.9–21.4–27.9(–40) × (3.0–)3.7–4.3–4.9(–5.4) μm, thin-walled, mainly cylindrical, apically usually obtuse, sometimes attenuated; contents with less heteromorphous-granulose, no color change in SV. Primordial hyphae near the pileus center are often smaller, always single-celled, thin-walled, (15–)16.4–21.7–27(–32) × (3.0–)3.4–3.9–4.4(–5.2) μm, clavate, apically typically obtuse or occasionally attenuated, contents with heteromorphous-granulose. **Cystidioid hyphae or oleiferous hyphae:** not observed.

**Habit and habitat:** Individual or scattered in broad-leaved forests such as *B. costata* and *P. davidiana* trees.

**Additional specimens examined:** CHINA, Hebei Province, Chengde City, Xinglong County, Baboziling, 40°18′36″ N, 117°35′6″ E, alt. 880 m, 20 August 2021, coll. C.-L.H., R.-T.Z. and G.-Q.C. (BJTC T2101).

**Notes:***Russula plana* belongs to subsection *Chamaeleontinae*. Phylogenetic analyses showed that *R. plana*, *R. olivascens* and *R. miyunensis* are closely related (Figure 1). Morphologically, *R. miyunensis* has light yellow to grayish-yellow, dark red and dark brown color in the pileus middle, colorless on the stipe, larger basidia (36.2–40.8 × 10.4–13.4 μm) than *R. plana*, hymenial cystidia on lamellae sides turning light brown in SV. *Russula olivascens* has light green-yellow pileus, bigger basidia (39–52 × 12–15 μm) than *R. plana* [57]. *Russula plana* is similar to *R. clavatohyphata,* which without molecular data, but *R. clavatohyphata* has short warty striate on the edge of the pileus, forked lamellae near the stipe, bigger basidia (22–54 × 9–13 μm) and smaller basidiospores (6.34–7.63 × 5.53–6.7 μm), hymenial cystidia on lamellae edges without color change in SV [30].

***Russula sinoparva* C. L. Hou, H. Zhou,** & **G. Q. Cheng, sp. nov.**

**Figure 11:**
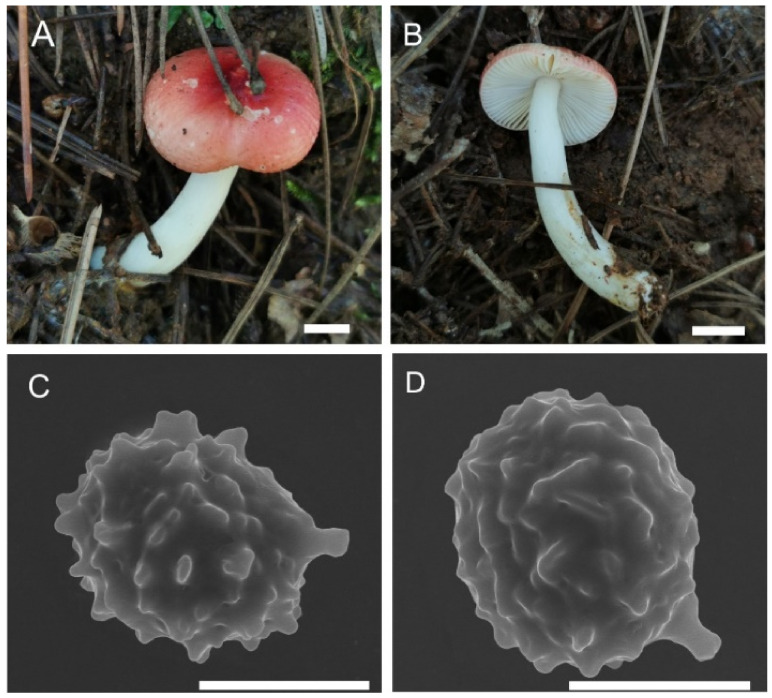
*Russula sinoparva* (BJTC ZH441). (**A**,**B**) Basidiomata. (**C**,**D**) Basidiospores. Scale bar: (**A**,**B**) = 10 mm, (**C**,**D**) = 5 μm.

**Figure 12:**
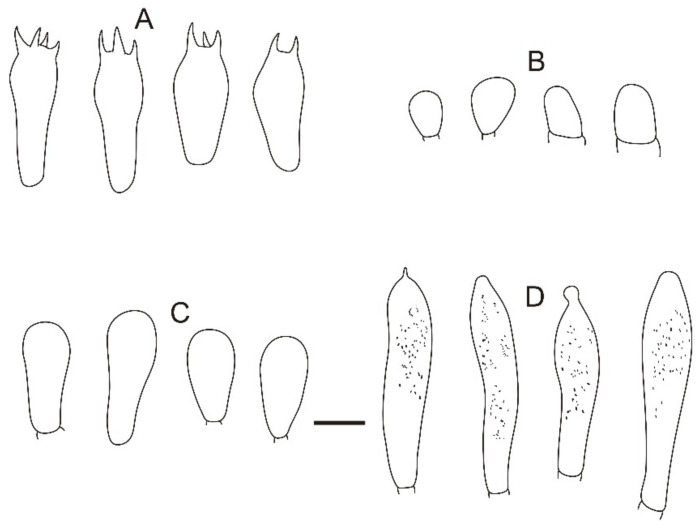
Microscopic features of *Russula sinoparva* (BJTC ZH441). (**A**) Basidia. (**B**) Basidiola. (**C**) Marginal cells. (**D**) Hymenial cystidia on lamellae sides. Scale bar: 10 μm.

**Figure 13:**
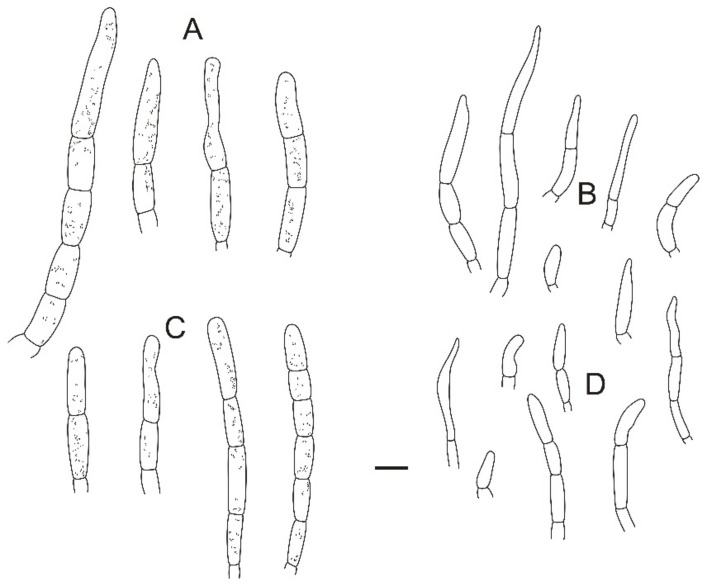
Microscopic features of *Russula sinoparva* (BJTC ZH441). (**A**) Pileocystidia near the pileus margin. (**B**) Hyphal terminations near the pileus margin. (**C**) Pileocystidia near the pileus center. (**D**) Hyphal terminations near the pileus center. Scale bar: 10 μm.

**Figure 14:**
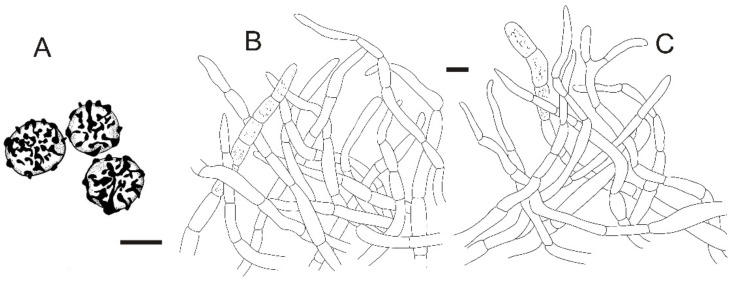
Microscopic features of Russula sinoparva (BJTC ZH441). (**A**) Basidiospores. (**B**) Hyphal terminations near the pileus margin. (**C**) Hyphal terminations near the pileus center. Scale bar: (**A**) = 5 μm; (**B**,**C**) = 10 μm.

Figure 11, Figure 12, Figure 13 and Figure 14.

**MycoBank: MB 845048**

**Diagnosis:***Russula sinoparva* is diagnosed by small basidiomata, light pink to pink pileus, subglobose to broadly ellipsoid basidiospores ornamented with small amyloid warts, the absence of hymenial cystidia on lamellae edges, bigger hymenialcystida on lamellae sides. *Russula sinoparva* and *Russula cessans* A. Pearson have similar morphological characteristics, but *R. sinoparva* has light pink to pink pileus and smaller basidiospore, and bigger hymenial cystidia on lamellae sides. 

**Holotype:** CHINA, Beijing, Huairou District, Erdaogou Village, 40°52′23.8″ N, 116°31′22.4″ E, alt. 758 m, 20 August 2019, coll. X.-Y.S., H.Z. and R.-T.Z. (BJTC Z441).

**Etymology:** The epithet “*sinoparva*“ refers to this Chinese species that has smaller basidiomata resembling the *Russula parva* Carteret & Reumaux.

**Basidiomata:** small size, pileus 18–30 mm in diameter, initially convex lenticular when young, flattened when mature, margin striations with small verrucas, sharp, cracked margin not obvious, light pink (#ffb6c1) to pink (#ffc0cb), central dark red (#ff0000) to strong deep red (#985144). **Lamellae:** white (#ffffff) to light yellow-brown (#ffffed), with 7–10 gills per cm at the edges, brittle, unequal, hardly forked. **Stipe:** 28–54 × 8–15 mm, white (#ffffff), sometimes brownish (#a52a2a), subcylindrical, smooth, firm, dilate gradually at the base. **Context:** 4–8 mm thick in half of the pileus radius, white (#ffffff) without color change when bruised. **Spore print:** not observed.

**Basidiospores:** (5.6–)6.5–7.0–7.6(–8.4) × (5.4–)5.8–6.3–6.7(–7.2) μm, [Q = (1.01–)1.04–1.13–1.21(–1.35)], subglobose to broadly ellipsoid, starchy ornamented, ornamentation of relatively small, moderately distant [(4–)5–7(–8) in a 3 μm diameter circle] amyloid warts, 0.3–1.0 μm high, occasionally to frequently fused in pairs or triplets, short-branched chains [(0–)1–3 (–4) in the circle], frequently connected by short or long, fine line connections [(0–)1–3 in the circle], suprahilar spot small. **Basidia:** (28–)29.6–34.5–39.4(–45) × (9–)10.5–11.8–13.1(–14) μm, 2–4-spored, clavate or fusiform, with particles and oil droplets, basidiola clavate or subcylindrical, ca. 5–15 μm wide. **Hymenial cystidia:** widely dispersed, ca. 200–300/mm^2^. **Hymenial cystidia on lamellae sides:** (30–)40.2–49.6–59.1(–67) × (7.2–)8.5–10–11.6(–13) μm, clavate or fusiform, apically often obtuse or mucronate, sometimes with 3–5-μm-long appendage, contents with refracted inclusions, or sometimes with granulose or crystalline, turning ash black (#0d0d0d) in SV. **Hymenial cystidia on lamellae edges:** not observed.

**Pileipellis:** orthochromatic in cresyl blue, sharply delimited from the underlying context, 70–100 μm deep, two-layered. Suprapellis 30–60 μm deep, strongly gelatinized, made up of ascending to erect, and slight interlaced hyphae. Subpellis 50–70 μm deep, composed of horizontally oriented, relatively dense, intricate, 3–6-μm-wide hyphae. Hyphal terminations near the pileus margin occasionally branched, sometimes flexuous, thin-walled; terminal cells (7.5–)14.7–23.5–32.4(–44) × (2.1–)2.6–3.4–4.1(–5.0) μm, mainly subcylindrical or clavate, apically mainly obtuse, occasionally attenuated, subterminal cells often wider, ca. 2–6 μm wide, always unbranched; hyphal terminations near the pileus center similar to those near the pileus margin; terminal cells (9–)16.7–31.9(–38) × (2.8–)3.1–4.3(–5.2) μm, mainly subcylindrical, occasionally ellipsoid, apically obtuse, constricted or attenuated; subterminal cells often wider, ca. 3–6 μm, always unbranched. Pileocystidia near the pileus margin are always 2–5-celled, a few one-celled, terminal cells (12–)18.1–32.6–47.2(–61) × (3.7–)4.7–5.9–7.2(–8.1) μm, thin-walled, mainly cylindrical or subcylindrical, occasionally clavate, apically usually obtuse, contents with granulose-heteromorphous, turning light ash black (#0d0d0d) in SV. Pileocystidia near the pileus center are often smaller, always 2–6-celled, thin-walled, terminal cells (12–)21.4–29.6–37.8(–45) × (4.8–)5.0–5.6–6.2(–7.2) μm, clavate or cylindrical, apically typically obtuse or occasionally attenuated, contents with granulose or occasionally crystalline. **Cystidioid hyphae:** in subpellis and context with heteromorphous-granulose contents, oleiferous hyphae in the subpellis close to the context.

**Habit and habitat:** Individual or scattered in coniferous forests and mixed coniferous and broad-leaved forests of *Pinus tabuliformis* Carr. and *Juglans mandshurica* Maxim. 

**Additional specimens examined:** CHINA, Beijing, Huairou District, Sunzhazi Village, 40°56′39.1″ N, 116°30′23.4″ E, alt. 780 m, 25 August 2020, coll. C.-L.H., R.-T.Z. and G.-Q.C. (BJTC C540).

**Notes:***Russula sinoparva* belongs to the subsection *Puellarinae* Singer. Phylogenetic analyses revealed that *R. sinoparva* is related to *R. odorata*, *R. khinganensi*s, and *R. subversatilis* (new species in this paper) (Figure 1 and Figure 2). Morphologically, they are somewhat similar in pileus shape and basidia size. However, *R. sinoparva* is diagnosed by the light pink to pink, central dark red to deep red pileus, strongly gelatinized suprapellis, 1–3-celled pileocystidia near the pileus margin; *R. odorata* is distinguished from *R. sinoparva* by the central brown or olive pileus and irregularly bifurcated lamellae. *R. khinganensis* by the livid brown or deep livid brown to russet vinaceous pileus and thicker pileipellis [24], and *R. subversatilis* by the light gray-red to deep red, central yellowish-brown to dark red pileus, bigger basidia and hymenial cystidia on lamellae sides. *Russula cessans,* which is without molecular data, is similar to *R. sinoparva* in lamellae color and density, but it has a pileus of black color in the middle and smooth, unstriped edges, bigger spores (8–9 × 7–8 µm) and smaller hymenial cystidia on lamellae sides (7–9 µm) [58].

***Russula sinorobusta* C. L. Hou, H. Zhou,** & **G. Q. Cheng, sp. nov.**

**Figure 15:**
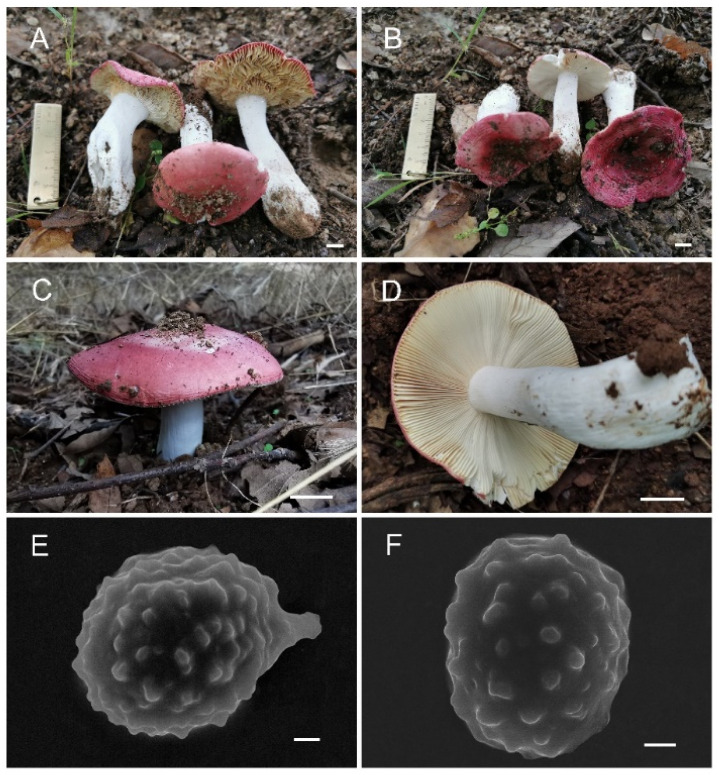
*Russula sinorobusta* (BJTC Z052). (**A**–**D**) Basidiomata. (**E**,**F**) Basidiospores. Scale bar: (**A**–**D**) = 10 mm, (**E**,**F**) = 1 μm.

**Figure 16:**
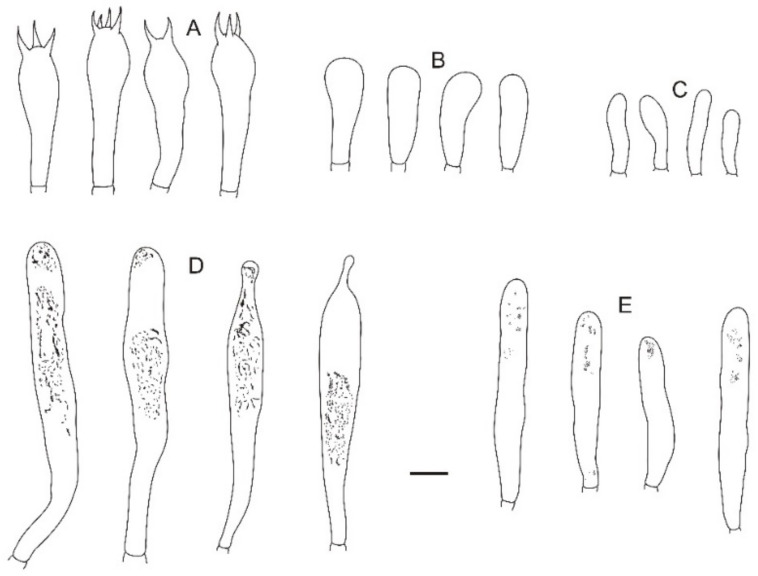
Microscopic features of *Russula sinorobusta* (BJTC Z052). (**A**) Basidia. (**B**) Basidiola. (**C**) Marginal cells. (**D**). Hymenial cystidia on lamellae sides. (**E**) Hymenial cystidia on lamellae edges. Scale bar: 10 μm.

**Figure 17:**
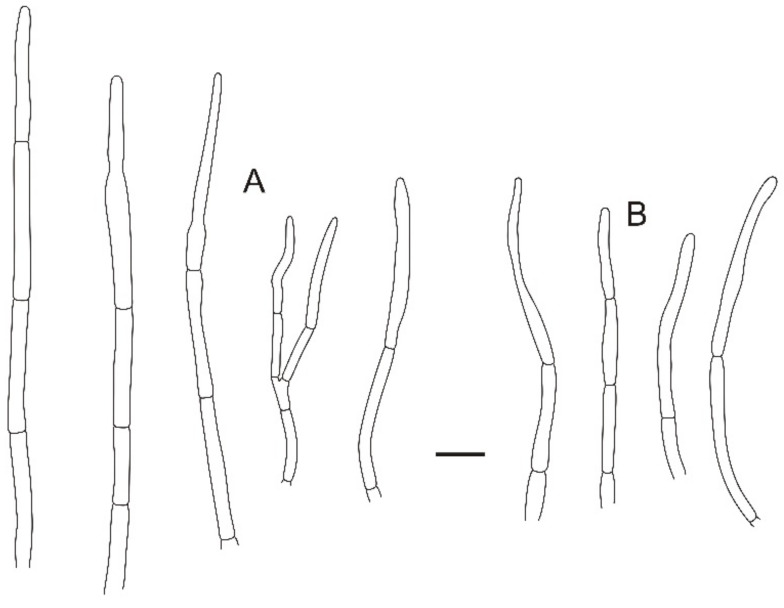
Microscopic features of *Russula sinorobusta* (BJTC Z052). (**A**) Hyphal terminations near the pileus margin. (**B**) Hyphal terminations near the pileus center. Scale bar: 10 μm.

**Figure 18:**
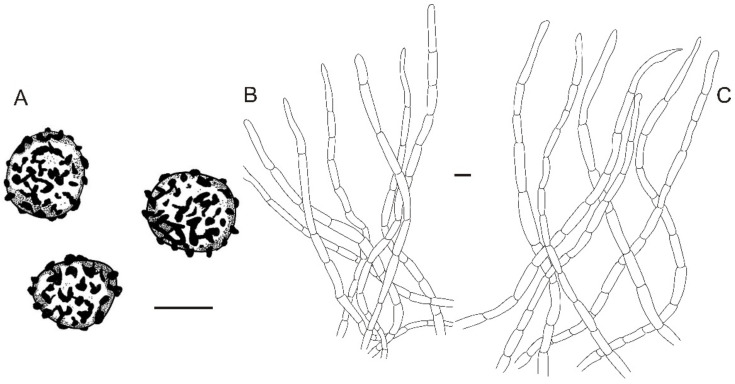
Microscopic features of *Russula sinorobusta* (BJTC Z052). (**A**) Basidiospores. (**B**) Hyphal terminations near the pileus margin. (**C**) Hyphal terminations near the pileus center. Scale bar: (**A**) = 5 μm; (**B**,**C**) = 10 μm.

Figure 15, Figure 16, Figure 17 and Figure 18.

**MycoBank: MB 845050**

**Diagnosis:***Russula sinorobusta* is diagnosed by small to medium-sized basidiomata, gray-red to rose red, central deep red pileus, basidiospores ornamented with small amyloid warts, suprahilar spot small, longer basidia, shorter hymenial cystidia on lamellae sides, pileocystidia absent. Morphologically, *R. sinorobusta* is similar to *R. intermedia* and *Russula vinosa* Lindblad., but *R. sinorobusta* has gray-red to rose red, central deep red pileus, and smaller basidiospore.

**Holotype:** CHINA, Beijing, Changping District, Yanshou Temple, 40°22′23.3″ N, 116°19′22.3″ E, alt. 270 m, 14 August 2019, coll. J.-Q.L. and H.Z. (BJTC Z052).

**Etymology**: The epithet “*sinorobusta*” refers to the stipe of this Chinese species that is relatively sturdy, resembling the *Russula robusta* R. Heim.

**Basidiomata**: small to medium size, pileus 51–82 mm in diameter, initially hemispherical when young, applanate with depressed in the center when mature, sometimes convex, slightly curved in edges, smooth when young, wrinkle in age, sticky when wet, peeling to 1/5 of the radius. gray-red (#8e6f70) to rose red (#b57281), sometimes deep red (#3b1f1f) in the center. **Lamellae:** white (#ffffff) to light yellow-brown (#ffffed), with 9–11 gills per cm at the edges, adnate, equal, lamellulae absent, hardly forked. **Stipe:** 60–102 × 19–34 mm, white (#ffffff), cylindrical, becoming hollow when mature, slightly inflated near the base, longitudinally striate. **Context:** 13–21 mm thick in half of the pileus radius, white (#ffffff), without color change when bruised. **Spore print:** not observed.

**Basidiospores:** (5.7–)6.2–6.6–7(–7.4) × (5.3–)5.5–5.9–6.3(–7.3) μm, [Q = (1.01–)1.06–1.12–1.18(–1.22)], subglobose to broadly ellipsoid, ornamentation of relatively of small, moderately distant to dense [(4–)5–8(–10)] in a 3 μm diameter circle] amyloid warts, 0.2–0.7 μm high, with abundant isolated verrucous, occasionally fused in pairs, triplets or short chains [0–2(–3) in the circle], occasionally connected by short, fine line connections [0–2(–3) in the circle], suprahilar spot small, weakly amyloid. **Basidia:** (35–)41.1–46–50.9(–57) × (11–)11.4–12.5–13.6(–14) μm, 2–4-spored, clavate or broadly ellipsoid, with particles and oil droplets, basidiola clavate or subcylindrical, ca. 6–10 μm wide. **Hymenial cystidia:** moderately numerous, ca. 720–900/mm^2^. **Hymenial cystidia on lamellae sides:** (60–)70.9–80.3–89.7(–94) × (10–)10.4–11.1–11.8(–12) μm, thin-walled, clavate or fusiform, apically mainly obtuse, occasionally mucronate or constricted, with 3–7-μm-long appendage; contents with granulose or banded, turning gray (#808080) to grayish purple in SV. **Hymenial cystidia on lamellae edges:** smaller and narrower to hymenial cystidia on lamellae sides, (40–)43.1–50–56.9(–63) × (6.0–)6.4–7.5–8.6(–10) μm, clavate or subcylindrical, apically often obtuse, sometimes with 2–4 μm long appendage, contents with granulose or banded.

**Marginal cells:** (14–)14.9–20.6–26.3(–33) × 5.8–6.5–7.2(–8.1) μm, cylindrical orclavate. **Pileipellis:** orthochromatic in cresyl blue, sharply delimited from the underlying context, 100–190 μm deep, two-layered. Suprapellis 50–100 μm deep, weakly gelatinized, composed of ascending to erect hyphae, trichoderm. Subpellis 45–90 μm deep, strongly gelatinized, composed of horizontally oriented, relatively dense, intricate, 3–9-μm wide hyphae. Hyphal terminations near the pileus margin occasionally branched, sometimes flexuous, thin-walled; terminal cells (14–)14.3–57.6(–62) × 2–4 μm, mainly cylindrical or subcylindrical, apically obtuse, subterminal cells often wider, ca. 2–3 μm wide, always unbranched; Hyphal terminations near the pileus center similar to those near the pileus margin; terminal cells (15–)15.6–45.3(–47.8) × (2–)3–5 μm, cylindrical, apically obtuse; subterminal cells often wider, always unbranched, ca. 2–3 μm wide. Pileocystidia not observed. **Cystidioid hyphae or oleiferous hyphae:** not observed.

**Habit and habitat:** Individual or scattered in broad-leaved forests of *Castanea mollissima* Blume.

**Additional specimens examined:** CHINA, Beijing, Changping District, Yanshou Temple, 40°22′23.3″ N, 116°19′22.5″ E, alt. 270 m, 14 August 2019, coll. J.-Q.L. and H.Z. (BJTC Z050); CHINA, Beijing, Changping District, Yanshou Temple, 40°22′7.4″ N, 116°19′21.7″ E, alt. 223 m, 26 July 2019, coll. G.-Q.C. and H.Z. (BJTC Z662).

**Notes:***Russula sinorobusta* belongs to subsection *Roseinae* Singer ex Sarnari. Multi-loci phylogenetic analysis showed that *R. sinorobusta*, *R. minutula* and *R. rosea* are closely related (Figure 1). *Russula sinorobusta* may be related to *Russula lepidicolor* Romagn. and *Russula intermedia* P. Karst., but not have supported value in ITS tree (Figure 2). Morphologically, *R. sinorobusta* is similar to *R. intermedia* and *R. vinosa*, but *R. vinosa* has a short and fuzzy striate on the edge of the pileus, the pileus middle color is copper, ochre, or brown, and the lamellae is close to the stipe forked, stipe hollow, larger basidiospores (8–11.5 × 6.5–8.5 μm) and hymenial cystidia on lamellae sides (85–120 × 10–13 μm) than *R. sinorobusta* [55]. *Russula intermedia* has wider hymenial cystidia on lamellae edges (9–11 μm) than *R. sinorobusta*, the width of pileocystidia is 4–11 μm, and with lilac color in SV [39].

***Russula subversatilis* C. L. Hou, H. Zhou,** & **G. Q. Cheng, sp. nov.**

**Figure 19:**
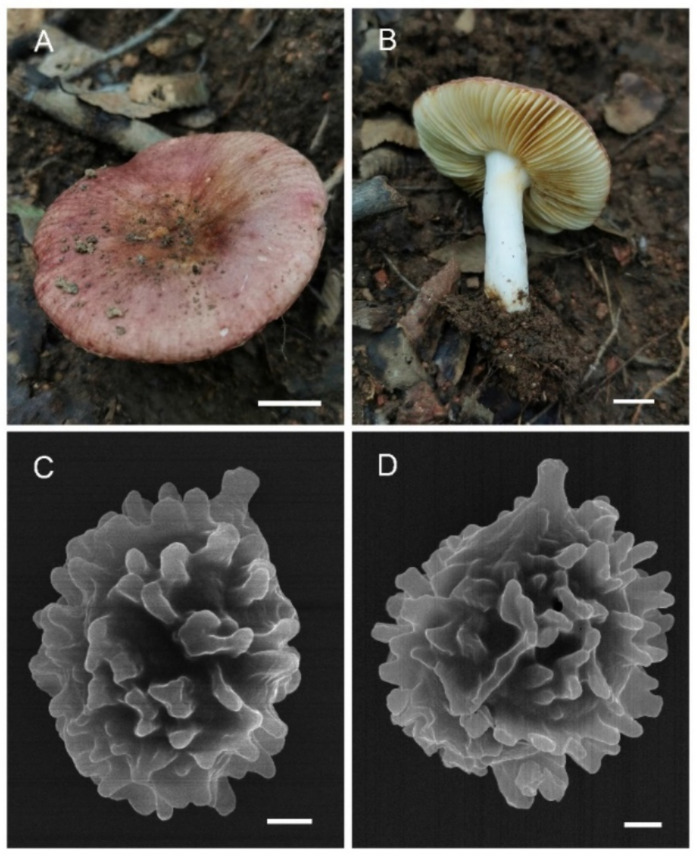
*Russula subversatilis* (BJTC C653). (**A**,**B**) Basidiomata. (**C**,**D**) Basidiospores. Scale bar: (**A**,**B**) = 10 mm, (**C**,**D**) = 1 μm.

**Figure 20:**
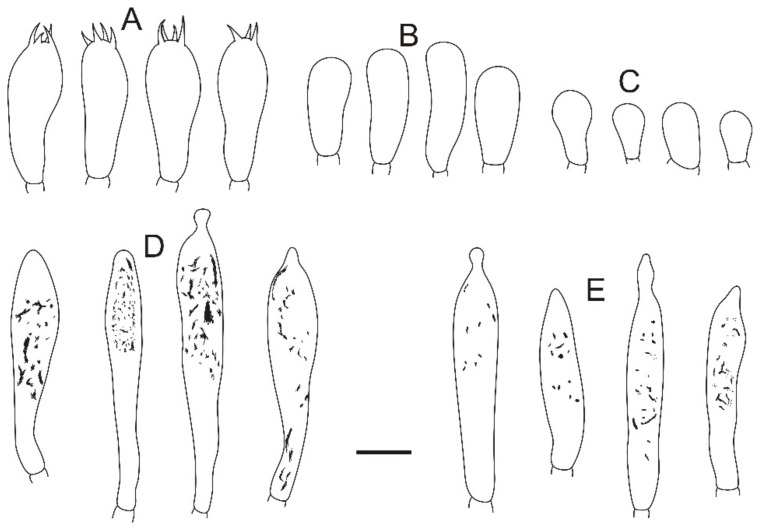
Microscopic features of *Russula subversatilis* (BJTC C653). (**A**) Basidia. (**B**) Basidiola. (**C**) Marginal cells. (**D**) Hymenial cystidia on lamellae sides. (**E**) Hymenial cystidia on lamellae edges. Scale bar: 10 μm.

**Figure 21:**
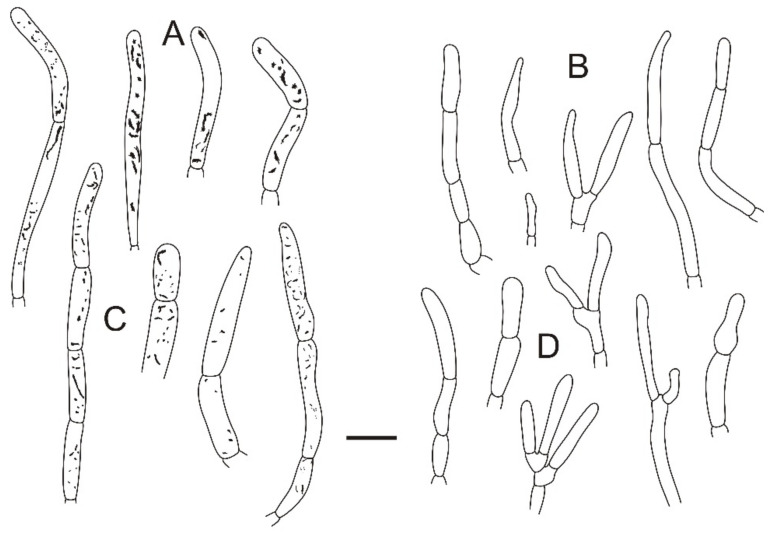
Microscopic features of *Russula subversatilis* (BJTC C653). (**A**) Pileocystidia near the pileus margin. (**B**) Hyphal terminations near the pileus margin. (**C**) Pileocystidia near the pileus center. (**D**) Hyphal terminations near the pileus center. Scale bar: 10 μm.

**Figure 22:**
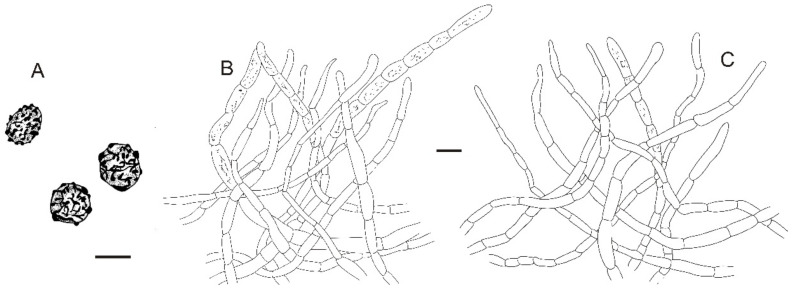
Microscopic features of *Russula subversatilis* (BJTC C653). (**A**) Basidiospores. (**B**) Hyphal terminations near the pileus margin. (**C**) Hyphal terminations near the pileus center. Scale bar: (**A**) = 5 μm; (**B**,**C**) = 10 μm.

Figure 19, Figure 20, Figure 21 and Figure 22.

**MycoBank: MB 845051**

**Diagnosis:***Russula subversatilis* is diagnosed by light gray-red to deep red, central yellowish-brown to dark red pileus, basidiospores ornamented with amyloid warts or spines, and more or less reticulate or chain-like, bigger basidia and hymenial cystidia on lamellae sides, light red pileocystidia in sulfovanillin. *Russula subversatilis* and *R. versatilis* Romagn have similar morphological characteristics, but *R. subversatilis* has light gray-red to deep red, central yellowish-brown to dark red pileus, shorter basidiospore, and narrower pileocystidia. 

**Holotype:** CHINA, Beijing, Miyun District, Heilongtan, 40°33′38.1″ N, 116°46′55.8″ E, alt. 255 m, 27 August 2020, coll. C.-L.H. and G.-Q.C. (BJTC C653).

**Etymology:** The epithet “*subversatilis”* refers to its morphological similarity to *Russula versatilis* Romagn.

**Basidiomata:** small to medium size, pileus 32–55 mm in diameter, initially hemispherical when young, flattened when mature, slightly concave in the middle, slightly curved in the margin, sticky when wet, with inconspicuous striations, margin light gray-red (#b09a95) to deep red (#985144), central yellowish-brown (#aa8d6f) to dark red (#481c1c). **Lamellae:** white (#ffffff) to light yellow-brown (#ffffed), with 5–7 gills per cm at the edges, free, brittle, lamellulae absent, hardly forked. **Stipe:** 35–65 × 15–20 mm, white (#ffffff), sometimes with brownish (#a52a2a) on the base, cylindrical, smooth, firm. **Context:** 8–14 mm thick in half of the pileus radius, white (#ffffff), without color change when bruised. Spore print: not observed.

**Basidiospores:** (6.1–)6.6–7.1–7.6(–8.2) × (5.3–)5.7–6.1–6.6(–7.3) μm, [Q = (1.02–)1.09–1.17–1.25(–1.32)], subglobose to broadly ellipsoid, ornamentation of relatively small, moderately distant to dense [(5–)6–9(–10) in a 3 μm diameter circle] amyloid warts or spines, 0.4–0.9 μm high, occasionally formed reticulate, occasionally to frequently fused in short or long branched chains [(0–)1–3 (–5) in the circle], occasionally connected by short or long, fine line connections [(0–)1–2(–3) in the circle], suprahilar spot small, amyloid. **Basidia:** 33.5–37–40.5(–45.2) × (11.8–)12.6–13.6–14.5(–15.2) μm, 2–4-spored, clavate, with particles and oil droplets, basidiola clavate or subcylindrical, ca. 9–14 μm wide. **Hymenial cystidia:** moderately numerous, ca. 950/mm^2^. **Hymenial cystidia on lamellae sides:** (47.6–)53.2–58.7–64.2 (–65.3) × (9.2–)10.5–11.9–13.3(–14.1) μm, clavate or subfusiform, apically mainly obtuse, often with 5–7-μm-long appendage, thin-walled; contents with heteromorphous-crystalline or granulose, mainly in the middle and upper part, turning purple (#800080) in SV. **Hymenial cystidia on lamellae edges:** similar to hymenial cystidia on lamellae sides, (36.5–)42.7–49.5–56.3(–64.4) × (6.9–)7.4–8.5–9.6(–10.1) μm, clavate or subfusiform, apically often obtuse, sometimes with 2–6-μm-long appendage, contents with granulose or crystalline, turning purple (#800080) in SV.

**Marginal cells:** (14.2–)15.6–17.8–20(–21.5) × (7.8–)8.4–9.2–10.1(–11.4) μm, usually broadly clavate and shorter than basidiola. **Pileipellis:** orthochromatic in cresyl blue, sharply delimited from the underlying context, 90–140 μm deep, two-layered. Suprapellis 40–60 μm deep, less gelatinized, composed of relatively loose, ascending to erect hyphae. Subpellis 60–100 μm deep, strongly gelatinized, composed of relatively dense, intricate, horizontally oriented near context, 2–7 μm wide hyphae. Hyphal terminations near the pileus margin occasionally branched, sometimes flexuous, thin-walled; terminal cells (12.4–)16.1–21.9–27.7(–32.4) × (2.0–)2.5–3.3–4.1(–5.1) μm, mainly cylindrical, sometimes clavate or fusiform, apically obtuse or constricted, subterminal cells often wider, ca. 2–4 μm wide, occasionally branched; hyphal terminations near the pileus center similar to those near the pileus margin; terminal cells (10.2–)12.9–23.5(–28.7) × (2.0–)2.3–4.1(–4.9) μm, cylindrical or subfusiform, apically obtuse, sometimes attenuated or constricted; subterminal cells often wider, occasionally branched, ca. 2–5 μm wide. Pileocystidia near the pileus margin are always 2–3 celled, sometimes one-celled, thin-walled, terminal cells (30.2–)35–67.8(–88.4) × (3.0–)3.5–5.3(–6.1) μm, cylindrical, subclavate or subfusiform, apically usually obtuse, sometimes attenuated, contents with abundant, heteromorphous, granulose or occasionally crystalline, turning light ash black (#0d0d0d) to light red (#ff4d4d) in SV. Pileocystidia near the pileus center are often smaller, always 2–3-celled, thin-walled, (21.4–)25.5–60.7(–78.1) × (3.8–)4.1–5.7(–6.0) μm, cylindrical or subclavate, apically obtuse, contents with heteromorphous, granulose**. Cystidioid and oleiferous hyphae:** not observed.

**Habit and habitat:** Individual in broad-leaved forests of *Carpinus turczaninowii*.

**Additional specimens examined:** CHINA, Beijing, Miyun District, Heilongtan, 40°33′38.2″ N, 116°46′56.2″ E, alt. 265 m, 27 August 2020, coll. C.-L.H. and G.-Q.C. (BJTC T2001).

**Notes:***Russula subversatilis* belongs to the subsection *Puellarinae*. The phylogenetic trees (Figure 1 and Figure 2) show that *R. subversatilis* is closely related to *Russula carpini* R. Girard & Heinem, *Russula khinganensis* G.J. Li & R.L. Zhao, and *Russula solaris* Ferd. & Winge, but *R. carpini* has light brownish purple or yellow pileus, bigger basidiospores (7–10 × 6.5–8 μm) [57], *R. khinganensis* has thinner basidia (35–43 × 10–11 μm), and hymenial cystidia on lamellae sides (51–65 × 6–9 μm), no hymenial cystidia on lamellae edges [24], *R. solaris* has pale orange to tinged yellowish pileus, bigger basidia (40–52 × 12–14 μm) and basidiospores (7.5–9 × 6.5–7.5 μm) [59]. Morphologically, *R. versatilis*, which is without molecular data, is similar to *R. subversatilis* in light gray-red pileus, light yellow-brown lamellae, and basidiospores with isolated warts or spines. However, *R. versatilis* has a pink or cream color in the middle pileus, longer basidiospores (7–8.5 × 5–6 µm), and wider pileocystidia (6–8 µm) [60].

***Russula yanshanensis* C. L. Hou, H. Zhou,** & **G. Q. Cheng, sp. nov.**

**Figure 23:**
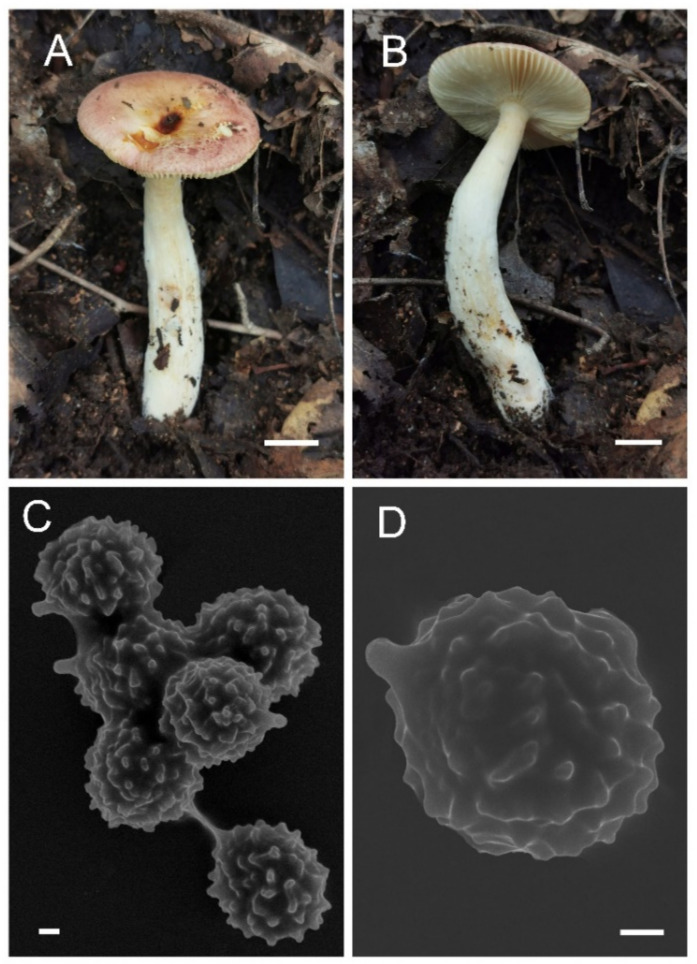
*Russula yanshanensis* (BJTC C561). (**A**,**B**) Basidiomata. (**C**,**D**) Basidiospores. Scale bar: (**A**,**B**) = 10 mm, (**C**,**D**) = 1 μm.

**Figure 24:**
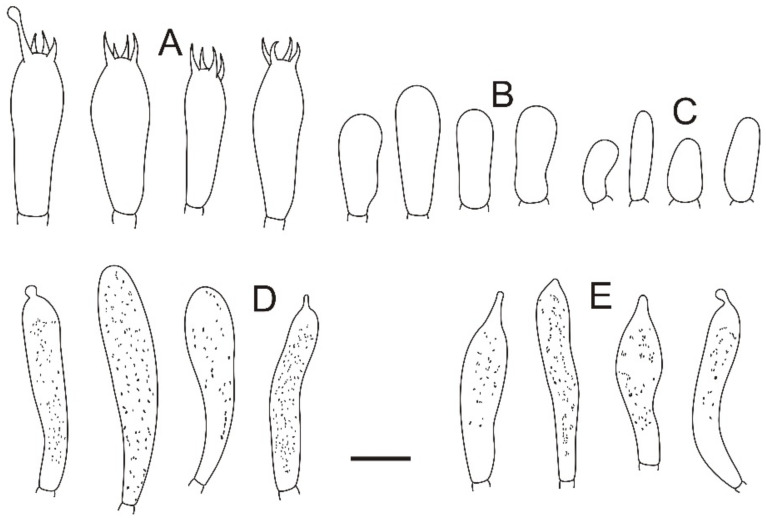
Microscopic features of *Russula yanshanensis* (BJTC C561). (**A**) Basidia. (**B**) Basidiola. (**C**) Marginal cells. (**D**) Hymenial cystidia on lamellae sides. (**E**) Hymenial cystidia on lamellae edges. Scale bar: 10 μm.

**Figure 25:**
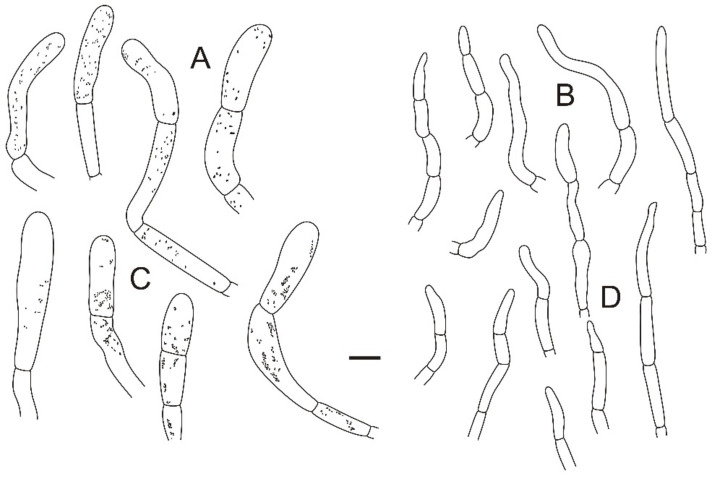
Microscopic features of *Russula yanshanensis* (BJTC C561). (**A**) Pileocystidia near the pileus margin. (**B**) Hyphal terminations near the pileus margin. (**C**) Pileocystidia near the pileus center. (**D**) Hyphal terminations near the pileus center. Scale bar: 10 μm.

**Figure 26:**
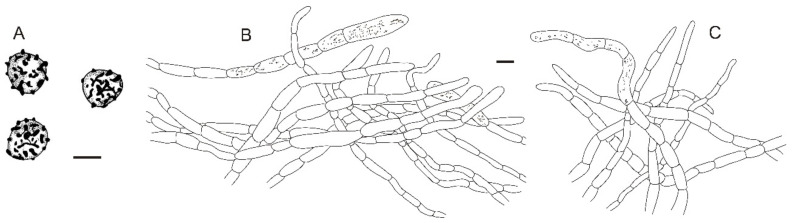
Microscopic features of *Russula yanshanensis* (BJTC C561). (**A**) Basidiospores. (**B**) Hyphal terminations near the pileus margin. (**C**) Hyphal terminations near the pileus center. Scale bar: (**A**) = 5 μm; (**B**,**C**) = 10 μm.

Figure 23, Figure 24, Figure 25 and Figure 26.

**MycoBank: MB 845052**

**Diagnosis:***Russula yanshanensis* diagnosed by light pink to pink, central light yellow to yellowish-brown pileus, smaller basidiospores ornamented with amyloid warts, and frequently chain-like, shorter hymenial cystidia on lamellae sides and hymenial cystidia on lamellae edges. *Russula yanshanensis* and *Russula cremeirosea* Murrill have similar morphological characteristics, but *R. yanshanensis* has pink, central light yellow to yellowish-brown pileus, shorter basidiospore, shorter hymenial cystidia on lamellae sides and hymenial cystidia on lamellae edges.

**Holotype:** CHINA, Beijing, Huairou District, Sunzhazi Village, 40°56′35″ N, 116°30′26″ E, alt. 763 m, 20 August 2019, coll. C.-L.H., J.-Q.L. and G.-Q.C. (BJTC C561).

**Etymology:** The epithet “*yanshanensis”* refers to the locality where the type specimen was collected.

**Basidiomata:** small to medium size, pileus 21–53 mm in diameter, initially hemispherical to convex when young, applanate with slightly depressed in the center when mature, not obvious striations or no striations in the margin, sticky when wet, peeling to 1/3 of the radius, margin light pink (#ffb6c1) to pink (#ffc0cb), sometimes red (#ff1a1a), central light yellow (#ffffed) to yellowish-brown (#aa8d6f). **Lamellae:** white (#ffffff) to light yellow-brown (#ffffed), with 6–9 gills per cm at the edges, free, brittle, unequal, lamellulae absent, hardly forked. **Stipe:** 31–60 × 10–20 mm, white (#ffffff), sometimes with brownish (#a52a2a) on the base, cylindrical, inflated toward the base, hollow, longitudinally striate. **Context:** 5–11 mm thick in half of the pileus radius, white (#ffffff), without color change when bruised. **Spore print:** not observed.

**Basidiospores:** (5.6–)6.1–6.6–7.1(–7.5) × (5.1–)5.5–5.8–6.1(–6.4) μm, [Q = (1.03–)1.07–1.14–1.21(–1.29)], subglobose to broadly ellipsoid, ornamentation of relatively small, dense [7–10(–11)] in a 3 μm diameter circle] amyloid warts, 0.2–0.6 μm high, occasionally with isolated warts, occasionally to frequently fused in short or long branched chains [(0–)1–3(–4) in the circle], frequently connected by short or long fine line connections [(0–)1–4(–5) in the circle], suprahilar spot not obvious, amyloid or weakly amyloid. **Basidia:** (34–)34.6–37.7–40.8(–47) × (11–)11.4–12.5–13.6(–15) μm, 2–4-spored, clavate, with particles and oil droplets, basidiola clavate or subcylindrical, ca. 8–13 μm wide. **Hymenial cystidia:** disperse, ca. 550/mm^2^. **Hymenial cystidia on lamellae sides:** (36–)37.5–50–62.5(–68) × (6.4–)7.1–8.3–9.5(–11) μm, thin-walled, clavate, subcylindricalor subfusiform, apically mainly obtuse, often with 2–4 μm long appendage; contents with heteromorphous-granulose or banded turning reddish brown (#a52a2a) in SV. **Hymenial cystidia on lamellae edges:** smaller to hymenial cystidia on lamellae sides, (30–)32.7–37.3–41.9(–45) × (7.0–)7.1–8–8.9(–9) μm, subclavate or fusiform, apically occasionally mucronate, sometimes with 2–7 μm long appendage, contents with heteromorphous-granulose or a few refractive, turning reddish brown (#a52a2a) in SV.

**Marginal cells:** (13.7–)14.6–17.3–19.9(–22.3) × (4.9–)5.7–6.5–7.2(–8.1) μm, cylindrical or ellipsoid. **Pileipellis:** orthochromatic in cresyl blue, sharply delimited from the underlying context, 50–80 μm deep, two-layered, less gelatinized. Suprapellis 40–60 μm deep, composed of ascending hyphae. Subpellis 20–35 μm deep, composed of horizontally oriented, intricate, 2–10 μm wide hyphae. Hyphal terminations near the pileus margin rarely branched, sometimes flexuous, thin-walled; terminal cells (13–)15.5–28.7–41.9(–52) × (2.0–)2.5–3.2–3.9(–4.1) μm, cylindrical, subfusiform, less lageniform, occasionally flexible, apically obtuse, sometimes attenuated or constricted, subterminal cells often wider, ca. 2–4 μm wide, always unbranched; hyphal terminations near the pileus center similar to those near the pileus margin; terminal cells (9.2–)10.4–19.4–28.4(–33.6) × (2.0–)2.2–3–3.8(–4.1) μm, mainly subcylindrical, less lageniform, apically obtuse; sometimes attenuated or constricted, subterminal cells often wider, always unbranched, ca. 2–4 μm wide. Pileocystidia near the pileus margin are always 2–3-celled, sometimes one-celled, thin-walled, sometimes subpellis extends to suprapellis, terminal cells (23.8–)25.6–39.4–53.2(–65.2) × (5.0–)5.4–7.6–8(–9.1) μm, cylindrical or clavate, apically usually obtuse; contents with abundant granulose or heteromorphous-crystalline, have weakly reaction turning light ash black (#0d0d0d) to light red (#ff4d4d) in SV. Pileocystidia near the pileus center simiar to the pileus margin, usually 2–3-celled, thin-walled, (20.2–)18.6–25.1–31.6(–39.8) × 6–6.7–9 μm, clavate, apically obtuse, contents with granulose or crystalline. **Cystidioid hyphae:** in subpellis and context with granulose or crystalline contents. Oleiferous hyphae in the subpellis.

**Habit and habitat:** Individual or scattered in coniferous or broad-leaved forests such as *P. tabuliformis* Carr., *Betula costata* Trautv. And *Populus davidiana* Dode trees.

**Additional specimens examined:** CHINA, Beijing, Huairou District, Sunzhazi Village, 40°56′40.5″ N, 116°30′25.2″ E, alt. 763 m, 20 August 2019, coll. C.-L.H., J.-Q.L. and G.-Q.C. (BJTC L349); CHINA, Beijing, Miyun District, Sileng Mountain, 40°28′23″ N, 117°6′17″ E, alt. 709 m, 30 August 2021, coll. C.-L.H., H.Z. and G.-Q.C. (BJTC Z1390); CHINA, Beijing, Miyun District, Sileng Mountain, 40°28′24″ N, 117°6′32″ E, alt. 663 m, 30 August 2021, coll. C.-L.H., H.Z. and G.-Q.C. (BJTC Z1385); CHINA, Beijing, Huairou District, Xiaozhuanghu Village, 40°52′35.6″ N, 116°31′16.6″ E, alt. 804 m, 20 August 2019, coll. H.Z. and X.-Y.S. (BJTC Z421); CHINA, Beijing, Huairou District, Sunzhazi Village, 40°56′35.6″ N, 116°30′26.0″ E, alt. 779 m, 25 August 2020, coll. C.-L.H., H.Z. and G.-Q.C. (BJTC C561); CHINA, Beijing, Yanqing District, Sijihuahai, 40°33′26″ N, 116°20′28″ E, alt. 733 m, 4 August 2021, coll. H.Z. and G.-Q.C. (BJTC Z1305); CHINA, Beijing, Yanqing District, Yudu Mountain, 40°33′5″ N, 115°52′15″ E, alt. 978 m, 31 August 2021, coll. C.-L.H., H.Z. and G.-Q.C. (BJTC Z1448).

**Notes:***Russula yanshanensis* belongs to the subsection *Puellarinae*. Specimens of *R. yanshanensis* were placed in a high-support branch on the phylogenetic tree and probably related to *R. puellaris* (Figure 1 and Figure 2). *Russula puellaris* can be distinguished from *R. puellaris* by its thinner basidiospores (28–43 × 9–12 µm), larger hymenial cystidia on lamellae sides (40–85 × 8–12), and turning gray in SV [55]. Morphologically, compare with similar species without molecular data. *Russula yanshanensis* is easy to be confused with *R. cremeirosea* in the appearance of basidiomata, but *R. cremeirosea* has bigger basidiospores (8–11 × 7.5–10 μm), longer hymenial cystidia on lamellae sides (50–76 × 3–10 µm), hymenial cystidia on lamellae edges (42–48 × 7–9 µm) and without pileocystidia [61]. 

## 4. Discussion

The topological structure of the two trees of the nrITS phylogenetic analysis (Figure 1) and the nrLSU-*rpb2*-*tef-1α*-mtSSU phylogenetic analysis (Figure 2) are basically similar, but the Bayesian posterior probability values and maximum likelihood bootstrap were higher in the nrLSU*-rpb2*-*tef-1α*-mtSSU analysis. In recent studies, many new species of *Russula* have been described only by nrITS loci phylogenetic analysis [14,25,62], which also results in the lack of other gene sequences and may cause some difficulties when performing multi-gene phylogenetic analysis. Therefore, it is crucial to discover better-differentiated DNA barcodes within the subgenus *Russula* in subsequent studies.

Through the current investigation, most species of the *Russula* were found in broad-leaved forests and associated with *Carpinus turczaninowii, Castanea mollissima*, *J. mandshurica*, *B. costata*, and *P. davidiana* trees, few species of the *Russula* occurred in coniferous forests, such as *P. tabuliformis*. The distributing characteristics may be related to the large area of broad-leaved forest in the Yanshan Mountains.

*Russula* Subgenus *Russula* has a very high species richness worldwide. At least 50 novel species have been described based on both morphological characters and molecular data since 2006, of which at least 30 species were reported from Asia [35]. In previous research, 16 species of *Russula* subgenus *Russula* are reported from southern China. [9,18,23,32,33,34,35,63,64]. Moreover, six species are from Northeast China [18,24,65,66]. Regarding the provincial distribution in China, more species of subgenus *Russula* are found in Guangdong Province and Heilongjiang Province. The reason for this phenomenon may be that these two provinces are actively investigated by mycologists. 

In this study, six new species belonged to three subsections under the subgenus *Russula*, namely subsection *Chamaeleontinae* (*R. miyunensis* and *R. plana*), subsection *Puellarinae* (*R. sinoparva, R. sinorobusta,* and *R. yanshanensis*) and subsection *Roseinae* (*R. subversatilis*). Subsection *Chamaeleontinae* belongs to species of normally small size. The cap is not much fleshy, the cuticle is detachable, and the margin is smooth or just grooved, especially when ripe; the stem is white, frail, and meaty, then hollow. Two new species, *R. miyunensis* and *R. plana,* described in this study, also fit these characteristics. Referring to Sarnari’s classification system, subsection *Chamaeleontinae* belongs to section *Amethystinae* Romagn. [5], and the position of this group in the systematic tree is also relatively stable. No Chinese *Russula* species belonging to subsection *Chamaeleontinae* have been identified in previous studies. Subsection *Puellarinae* was established by Singer in 1932 [67]. Singer had studied American and tropical *Russulas* prior to proposing his classification. Subsections *Chamaeleontina* Singer, *Subcompactinae* Singer, and *Puellarinae* were separated out within section *Constantes* Singer [68]. *Russula khinganensis,* described by Li et al., is also a member of subsection *Puellarinae* [24]. Subsection *Roseinae* was recognized by Sarnari in 1998 [5]. Morphologically, members of the subsection *Roseinae* provide a persistent bright red-colored reaction in dried fruit bodies with sulfovanilin. No species of subsection *Roseinae* were reported before among *Russula* species in China.

*Russula* contains a number of wild edible fungi in the world, and there are also a certain number of poisonous fungi. The classification of *Russula* is a difficult point in the classification of macrofungi [11]. Therefore, a classification study of the *Russula* is also essential to promote the study of edible species within the genus. According to incomplete statistics, 128 species of *Russula* are used as edible mushrooms in 28 countries worldwide [69]. Moreover, 78 edible species of *Russula* in China were recorded by Wu et al. [12], e.g., *Russula delica* Fr., *Russula densifolia* Secr. ex Gillet, *Russula griseocarnosa* X.H. Wang, Zhu L. Yang, & Knudsen. 

Through literature review, all three subsections in which the six new species of this study are located have edible species distributed in China [70]. In subsection *Chamaeleontinae*, *Russula turci* Bres. and *Russula roseipes* Secr. ex Bres. are representative species. In subsection *Puellarinae*, *Russula puellaris* Fr. is a representative species and is more widely distributed in China. In subsection *Roseinae*, there are more edible mushrooms known in this subsection, including *Russula pseudointegra* Arnould & Goris, *Russula rosea* Pers., *Russula lepidicolor* Romagn., and all the above species are also distributed in China. Therefore, the six new species discovered this time should also contain edible species that worth to be studied.

This study is the first report of the species of *Russula* subgenus *Russula* from the Yanshan Mountains in northern Beijing and northern Hebei Province. Considering the large area of China and its diverse forest types, it is reasonable to infer that many more species of the genus *Russula* are expected to be found in the following studies. On the premise of determining the number of *Russula* species, relevant studies on edible species can be further deepened.

## Figures and Tables

**Table 1:** Sequences information used in the nrLSU-*rpb2*-*tef-1α*-mtSSU phylogenetic analysis in this study.

Taxa	Voucher	Location	GenBank Accession Numbers			
			nrLSU	*mtSSU*	*rpb2*	*tef-1α*
*Multifurca aurantiophylla*	644/BB 09.119	New Caledonia	KU237581	KU237429	KU237867	KU238008
*Multifurca ochricompacta*	580/BB 07.010	USA	KU237565	KU237413	KU237851	KU237994
*Russula abbottabadensis*	LAH 310071	Pakistan	MN518356	MG386719	MG386737	MZ364137
*Russula abbottabadensis*	FH 00304558	Pakistan	MN518355	MG386721	MG386738	MZ364138
*Russula acrifolia*	543/BB 08.662	Italy	KU237535	KU237381	KU237821	KU237965
*Russula adusta*	223/BB 06.562	Canada	KU237476	KU237320	KU237762	KU237907
*Russula amethystina*	529/BB 07.314	Slovakia	KU237521	KU237367	KU237807	KU237951
*Russula archaeosuberis*	1118/BB 12.085	Italy	KU237593	KU237441	KU237878	KU238019
*Russula ayubiana*	LAH 35438	Pakistan	MZ358816	MZ364121	MZ364131	MZ364139
*Russula ayubiana*	LAH 35439	Pakistan	MZ358817	MZ364122	MZ364132	MZ364140
*Russula azurea*	537/BB 08.668	Italy	KU237529	KU237375	KU237815	KU237959
*Russula betularum*	BPL269	USA	KT933829	KT933969	KT933900	-
*Russula bicolor*	HMJAU 32180	China	KX095080	KX095031	-	-
*Russula brevipes*	226/BB 06.508	Mexico	KU237479	KU237323	KU237765	-
*Russula burlinghamiae*	548/BB 05.108	USA	KU237540	KU237386	KU237826	KU237970
*Russula carpini*	551/BB 07.262	Slovakia	KU237543	KU237389	KU237829	KU237973
*Russula chloroides*	572/BB 07.209	Slovakia	KU237559	KU237407	KU237845	KU237990
*Russula compacta*	228/BB 06.295	USA	KU237480	KU237324	KU237766	-
*Russula corallina-*	229/BB 06.324	USA	KU237481	KU237325	KU237767	KU237910
*Russula crustosa*	BPL265	USA	KT933826	-	KT933898	-
*Russula cuprea*	565/BB 07.233	Slovakia	KU237555	KU237401	KU237841	KU237984
*Russula decolorans*	549/BB 07.322	Slovakia	KU237541	KU237387	KU237827	KU237971
*Russula emetica*	635/JMT39-08092228	France	KU237578	KU237426	KU237864	-
*Russula exalbicans*	584/BB 07.786	France	KU237568	KU237416	KU237854	KU237996
*Russula farinipes*	576/BB 08.632	Italy	KU237561	KU237409	KU237847	KU237992
*Russula fattoensis*	Buyck 02.227	USA	MN315514	MN315537	MN326797	MN326800
*Russula fragilis*	443/BB 07.791	France	KU237506	KU237351	KU237792	-
*Russula glutinosa*	Roody WRWV 04.1154	USA	MN315511	MN315532	MN326798	MN326799
*Russula gracillima*	441/BB 07.785	France	KU237504	KU237349	KU237790	KU237934
*Russula griseobrunnea*	JAC11227	New Zealand	MW683630			
*Russula herrerae*	239/BB 06.532	Mexico	KU237486	KU237330	KU237772	KU237915
*Russula integra*	518/BB 07.198	Slovakia	KU237513	KU237359	KU237799	KU237943
*Russula laeta*	519/BB 07.267	Slovakia	KU237514	KU237360	KU237800	KU237944
*Russula laricina*	575/BB 08.681	Italy	KU237560	KU237408	KU237846	KU237991
*Russula leucomarginata*	RITF3133	China	MW309327	MW309338	MW310568	-
*Russula leucomarginata*	RITF3123	China	MW309328	MW309339	MW310569	-
*Russula lilacea*	435/BB 07.213	Slovakia	KU237498	KU237343	KU237784	KU237928
*Russula mansehraensis*	HUP SUR 180	Pakistan	MG944280	MG944266	MG944255	-
*Russula mansehraensis*	HUP SUR 803	Pakistan	-	MG944267	MG944256	-
*Russula minutula*	539/BB 08.636	Italy	KU237531	KU237377	KU237817	KU237961
***Russula miyunensis***	**BJTC Z1357**	**China**	-	**OP135984**	**OP156826**	**OP156837**
***Russula miyunensis***	**BJTC Z1355**	**China**	**OP133232**	**OP135985**	**OP156827**	-
*Russula mustelina*	1176/SA 09.88	Slovakia	KU237596	KU237444	KU237881	KU238022
*Russula nauseosa*	588/BB 07.285	Italy	KU237572	KU237420	KU237858	KU238000
*Russula nigricans*	429/BB 07.342	Slovakia	KU237495	KU237339	KU237781	KU237924
*Russula nothofagineae*	723/BB 09.044	New Caledonia	KU237583	KU237431	-	KU238010
*Russula nothofagineae*	726/BB 09.069	New Caledonia	KU237585	KU237433	KU237870	KU238012
*Russula odorata*	526/BB 07.186	Slovakia	KU237518	KU237364	KU237804	KU237948
*Russula olivascens*	530/BB 08.663	Italia	KU237522	KU237368	KU237808	KU237952
*Russula olivobrunnea*	JV28388	Finland	-	MW633232	-	-
***Russula plana***	**BJTC Z1398**	**China**	**OP133233**	**OP135986**	**OP156828**	**OP156838**
***Russula plana***	**BJTC T2101**	**China**	**OP265903**	**OP265901**	**OP267556**	**OP267558**
*Russula pseudoaurantiophylla*	740/BB 09.219	New Caledonia	KU237591	KU237439	KU237876	KU238017
*Russula puellaris*	523/BB 07.311	Slovakia	KU237515	KU237361	KU237801	KU237945
*Russula purpureoverrucosa*	GDGM32902	China	MG214699	-	MT085652	MT085623
*Russula quercus-floribundae*	LAH 36219	Pakistan	MN513043	MN053397	MN053389	MZ364152
*Russula quercus-floribundae*	LAH 36220	Pakistan	MN513043	MN053396	MN053390	MZ364153
*Russula raoultii*	561/BB 08.674	Italy	KU237551	KU237397	KU237837	KU237980
*Russula rosea*	430/BB 07.780	France	KU237496	KU237340	KU237782	KU237925
*Russula roseola*	RITF3418	China	MW309319	MW309330	MW310560	-
*Russula roseola*	RITF3428	China	MW309320	MW309331	MW310561	-
***Russula sinoparva***	**BJTC C540**	**China**	**OP133234**	**OP135987**	**OP156829**	**OP156839**
***Russula sinoparva***	**BJTC Z441**	**China**	**OP133235**	**OP135988**	-	**OP156840**
***Russula sinorobusta***	**BJTC Z050**	**China**	**OP133236**	**OP135989**	**OP156830**	**OP156841**
***Russula sinorobusta***	**BJTC Z052**	**China**	-	**OP135990**	-	**OP156842**
***Russula sinorobusta***	**BJTC Z662**	**China**	**OP133237**	**OP135991**	**OP156831**	**OP156843**
*Russula sichuanensis*	ZRL20162017	China	MG786572	MG792323	-	MG812160
*Russula solaris*	559/BB 07.282	Slovakia	KU237549	KU237395	KU237835	KU237978
*Russula* sp.	735/BB 09.172	New Caledonia	KU237588	KU237436	KU237873	KU238015
*Russula subsanguinaria*	RITF2236	China	MW309322	MW309333	MW310563	-
*Russula subsanguinaria*	RITF2208	China	MW309323	MW309334	MW310564	-
*Russula subtilis*	536/BB 05.107	USA	KU237528	KU237374	KU237814	KU237958
***Russula subversatilis***	**BJTC C653**	**China**	**OP133238**	**OP135992**	**OP156832**	**OP156844**
***Russula subversatilis***	**BJTC T2001**	**China**	**OP265904**	**OP265902**	**OP267557**	**OP267559**
*Russula turci*	528/BB 07.328	Slovakia	KU237520	KU237366	KU237806	KU237950
*Russula versicolor*	589/BB 07.288	Slovakia	KU237573	KU237421	KU237859	KU238001
*Russula vinosobrunneola*	HMAS 281138	China	MG786569	MG792320	-	MG812157
*Russula vinosobrunneola*	HMAS 278885	China	MG786570	MG792321	-	MG812158
***Russula yanshanensis***	**BJTC C561**	**China**	**OP133239**	**OP135993**	-	**OP156845**
***Russula yanshanensis***	**BJTC Z1448**	**China**	**OP133240**	**OP135994**	**OP156833**	-
***Russula yanshanensis***	**BJTC Z421**	**China**	**OP133241**	**OP135995**	-	**OP156846**
***Russula yanshanensis***	**BJTC Z1385**	**China**	**OP133242**	**OP135996**	-	**OP156847**
***Russula yanshanensis***	**BJTC Z1305**	**China**	**OP133243**	**OP135997**	**OP156834**	**OP156848**
***Russula yanshanensis***	**BJTC Z1390**	**China**	**OP133244**	**OP135998**	**OP156835**	**OP156849**
***Russula yanshanensis***	**BJTC L349**	**China**	**OP133245**	**OP135999**	**OP156836**	**OP156850**
*Russula zvarae*	538/BB 08.639	Italy	KU237530	KU237376	KU237816	KU237960

Remarks: The new generated sequences are emphasized in bold, “-” show no sequence. Database.

## Data Availability

All sequence data are available in NCBI GenBank following the accession numbers in the manuscript.
